# Downregulated CLIP3 induces radioresistance by enhancing stemness and glycolytic flux in glioblastoma

**DOI:** 10.1186/s13046-021-02077-4

**Published:** 2021-09-06

**Authors:** Hyunkoo Kang, Sungmin Lee, Kyeongmin Kim, Jaewan Jeon, Seok-Gu Kang, HyeSook Youn, Hae Yu Kim, BuHyun Youn

**Affiliations:** 1 Department of Integrated Biological Science, Pusan National University, Busan, Republic of Korea; 2 Present address: Institute of Bioinnovation Research, Kolon Life Science, Seoul, Republic of Korea; 3 Department of Radiation Oncology, Haeundae Paik Hospital, Inje University College of Medicine, Busan, Republic of Korea; 4 Department of Neurosurgery, Brain Tumor Center, Severance Hospital, Yonsei University College of Medicine, Seoul, Republic of Korea; 5 Department of Medical Sciences, Yonsei University Graduate School, Seoul, Republic of Korea; 6 Department of Integrative Bioscience and Biotechnology, Sejong University, Seoul, Republic of Korea; 7 Department of Neurosurgery, Haeundae Paik Hospital, Inje University College of Medicine, Busan, Republic of Korea; 8 Department of Biological Sciences, Pusan National University, Busandaehak-ro 63beon-gil 2, Geumjeong-gu, Busan, 46241 Republic of Korea

**Keywords:** CLIP3, Glimepiride, Glioblastoma, Glioblastoma stem-like cells, Radioresistance

## Abstract

**Background:**

Glioblastoma Multiforme (GBM) is a malignant primary brain tumor in which the standard treatment, ionizing radiation (IR), achieves a median survival of about 15 months. GBM harbors glioblastoma stem-like cells (GSCs), which play a crucial role in therapeutic resistance and recurrence.

**Methods:**

Patient-derived GSCs, GBM cell lines, intracranial GBM xenografts, and GBM sections were used to measure mRNA and protein expression and determine the related molecular mechanisms by qRT-PCR, immunoblot, immunoprecipitation, immunofluorescence, OCR, ECAR, live-cell imaging, and immunohistochemistry. Orthotopic GBM xenograft models were applied to investigate tumor inhibitory effects of glimepiride combined with radiotherapy.

**Results:**

We report that GSCs that survive standard treatment radiation upregulate Speedy/RINGO cell cycle regulator family member A (Spy1) and downregulate CAP-Gly domain containing linker protein 3 (CLIP3, also known as CLIPR-59). We discovered that Spy1 activation and CLIP3 inhibition coordinately shift GBM cell glucose metabolism to favor glycolysis via two cellular processes: transcriptional regulation of CLIP3 and facilitating Glucose transporter 3 (GLUT3) trafficking to cellular membranes in GBM cells. Importantly, in combination with IR, glimepiride, an FDA-approved medication used to treat type 2 diabetes mellitus, disrupts GSCs maintenance and suppresses glycolytic activity by restoring CLIP3 function. In addition, combining radiotherapy and glimepiride significantly reduced GBM growth and improved survival in a GBM orthotopic xenograft mouse model.

**Conclusions:**

Our data suggest that radioresistant GBM cells exhibit enhanced stemness and glycolytic activity mediated by the Spy1-CLIP3 axis. Thus, glimepiride could be an attractive strategy for overcoming radioresistance and recurrence by rescuing CLIP3 expression.

**Supplementary Information:**

The online version contains supplementary material available at 10.1186/s13046-021-02077-4.

## Background

Glioblastoma Multiforme (GBM) remains the most aggressive and non-curative malignant primary brain tumor in adults [1]. Current therapy entails surgical resection followed by radiotherapy with chemotherapy to eliminate highly proliferating tumor cells [2]. Median survival is approximately 15 months, and the overall survival rate has not significantly improved over the past 20 years [3]. Recently, GBM was classified into four subtypes (classical, neural, proneural, and mesenchymal) based on genetic and clinical profiles [4]. Although this knowledge helps predict prognosis of patients and response to therapy, personalized treatments or novel therapeutic curative strategies are urgently needed. At a cellular level, glioblastomas are viewed as hierarchies with glioblastoma stem-like cells (GSCs) capable of self-renewal and tumorigenic capacity at the apex, and giving rise to differentiated tumor cell types [5]. GSCs are defined by sustained proliferation, sphere formation capability, multilineage differentiation, rewired metabolism, and resistance to cytotoxic therapies including ionizing radiation (IR) [6]. However, conventional therapies rarely impact on the stemness of GSCs, but instead boosts their preferential survival and adaptation of stem-like cell properties by non-GSCs [7, 8]. Therefore, therapy-enriched GSCs contribute not only to tumor growth but also to tumor recurrence after chemoradiotherapy. Although targeting GSCs holds great promise as a therapeutic strategy for eradicating GBM, identifying cellular mechanisms that eliminate GSCs while sparing healthy cells and tissues has been met with little success to date, and no such drug are available in clinical practice [6, 9].

Speedy/RINGO cell cycle regulator family member A (Spy1), a member of Speedy/RINGO family, was reported to regulate GSCs division by directly binding to and activating cyclin-dependent kinases (CDKs), independently of phosphorylation, and bypassing cell cycle checkpoints [10]. A recent study showed that Spy1 expression was significantly elevated in GBM relative to low-grade glioma tissues, suggesting that Spy1 might contribute to processes that increase GSC populations in higher-grade gliomas [10, 11]. According to the study, Spy1 interacted with CLIPR59/CLIP3 (CAP-Gly domain containing linker protein 3, hereafter referred to as CLIP3), a membrane-associated protein with several protein-protein interaction domains reported to regulate glucose homeostasis [12]. In addition, the Spy1/CLIPR-3 interaction was proposed to confer resistance to TNF-α-induced apoptosis in GBM, a plausible mechanism by which GSCs could escape cell death induced by IR. Beyond negative correlation between CLIP3 and Spy1 expression in GBM tissues, few studies provide insight into how their interaction affect cellular processes. A recent study found that CLIP3 via TBC1 domain family member 4 (TBC1D4) modulates membrane translocation of Glucose transporter 4 (GLUT4) in adipocytes, pointing to a role in dynamic intracellular transport to the cell membrane [13]. Although CLIP3 is mainly expressed in the human brain, its relationship with brain glucose transporter GLUT1 and GLUT3 has not been studied. Given the molecular connections reported in the literature, and the role of Spy1 in GSC cell division, we hypothesized that a Spy1-CLIP3 axis could play important roles in GBM malignancy by regulating GSC proportion and GSC-specific glucose metabolism.

Cancer cells and especially cancer stem cells (CSCs) highjack control of metabolism to favor aerobic glycolysis rather than oxidative phosphorylation for ATP generation, which is known as the Warburg effect [14]. The metabolic reprogramming enables cancer cells to produce biological building blocks required for cell proliferation [15]. In brain cancer, the Warburg effect is considered more important because the brain is mostly fueled by glucose [16]. In GBM, several drugs that selectively target general metabolic pathways have been tested in clinical trials, while only a small proportion of the drugs regulate glucose metabolism with clinical significance [17]. Our previous study found increased glucose uptake in highly glycolytic GBM cells, consistent with the low ATP production efficiency of glycolysis leading to cellular increase GLUT expression to achieve a higher glucose uptake [18]. The major GLUT in GBM is GLUT1, but GSCs also express GLUT3, which has five times higher affinity for glucose than GLUT1, to adapt to high glucose demands [19]. Previously, glucose uptake was thought to be regulated by GLUT expression, but our understanding of how regulated cell surface trafficking impacts dynamics of glucose uptake is increasing [20, 21]. Translocation of GLUT1 to the plasma membrane is primarily controlled by autophagy-dependent recycling and whereas that of GLUT3 is controlled by the ras-related protein Rab-11A (Rab11a), which is the only known marker of its trafficking, to form Rab11a-positive recycling endosome [22, 23]. However, how membrane translocation of GLUTs might contribute to GBM etiology and recurrence remains unknown.

Temozolomide (TMZ), a first-line treatment for GBM, alkylates DNA bases and leads to DNA mismatch, inhibition of DNA replication, and cytotoxicity in highly proliferating cells [24]. However, approximately 50% of TMZ-treated GBM patients do not respond to TMZ because their tumors overexpressed O_6_-Methylguanine-DNA Methyltransferase (MGMT), and most patients experience serious side effects including bone marrow suppression and female infertility [25–27]. Despite these limitations, TMZ is the only drug that improves survival in GBM when combined with radiotherapy [28]. Although many studies have suggested novel molecular targets for GBM therapy, most drugs have clinically failed due to high toxicity or lack of efficacy [29]. Given the timelines and costs associated with bringing a new drug to the clinic, drug repositioning is emerging as a strategy for drug discovery [30]. Drugs that have demonstrated safety in patients, but failed efficacy endpoints in a specific indication, might become attractive therapeutic strategies if the existing drugs can specifically target biomarkers in other diseases. Based on this background, we hypothesized that a CLIP3-activating drug which has already been approved by the FDA successfully targets self-renewal and metabolic pathway mechanism of GBM, distinct from the current treatment approaches which solely target cell proliferation.

## Materials and methods

### Chemicals, antibodies, and reagents

Glimepiride and glibenclamide were obtained from Tokyo Chemical Industry (Tokyo, Japan). Antibodies specific for Spy1 and CLIP3 were purchased from Abcam (Cambridge, MA, USA); antibodies specific for β-actin, HSP90, CDK2, HA-probe, and GLUT3 were purchased from Santa Cruz Biotechnology (Santa Cruz, CA, USA); an antibody specific for CD133-PE were purchased from Miltenyi Biotec (Bergisch Gladbach, Germany). Phosphate Buffered Saline (PBS), Hank’s Balanced Salt Solution (HBSS), Eagle’s Minimum Essential Medium (MEM), Dulbecco’s Modified Eagle Medium/Nutrient Mixture F-12 (DMEM/F-12), fetal bovine serum (FBS), B27 Supplement (minus vitamin A), basic fibroblast growth factor (bFGF), epidermal growth factor (EGF), L-glutamine, sodium pyruvate, penicillin, streptomycin, and Trizol were purchased from Thermo Fisher Scientific (Cleveland, OH, USA). Collagenase D and DNase I recombinant were purchased from Sigma-Aldrich (St. Louis, MO, USA). Control siRNA and siRNA specific for Spy1, CLIP3, NRF1, CDK2, CD133, NES, and Kir6.2 were purchased from Bioneer (Daejeon, Republic of Korea). pGL3-NFAT luciferase vector was purchased from Addgene (Watertown, MA, USA); a plasmid for NRF1 was purchased from Origene (Rockville, MD, USA); a plasmid for Spy1-HA was purchased from Nova Lifetech (Singapore); plasmids for CLIP3, Rab11a-OFP, and GLUT3-GFP were purchased from Sino Biological (Beijing, China).

### Glioblastoma stem cell derivation

Patient-derived GSC11 glioblastoma stem cells were provided by Dr. Frederick F. Lang (Department of Neurosurgery, The University of Texas, M. D. Anderson Cancer Center, Houston, USA); patient-derived BCL20-HP01 and BCL20-HP02 glioblastoma stem cells were obtained from patients undergoing resection in accordance with a protocol approved by Haeundae Paik Hospital (Inje University, Busan, Republic of Korea); patient-derived TS19–176 glioblastoma stem cells were transferred via a material transfer agreement from Severance Hospital. BCL20-HP01 GSCs were derived from a GBM from a 47-year old male patient. BCL20-HP02 GSCs were derived from a GBM from a 38-year old male patient. Detailed information of the patients is summarized in Table S1. More specifically, after the resection, about 200 to 500 mg of tumor samples were collected into a tube containing DMEM/F-12 supplemented with B27. Tumor specimen was then washed with 5 ml of HBSS to remove blood and debris. After the washing, the tumor was cut into small fragments and minced with a sterile scalpel blade into approximately 1 mm^3^ fragment. To dissociate GBM tumor tissue, the minced tumor was treated with collagenase D (1 mg/ml) and DNase I (0.1 mg/ml) in HBSS and incubated at 37 °C for 30 to 90 min with gentle mixing. Finally, the solution was passed through the 70 μm sterile mesh filter to remove any large, undigested tumor pieces. To culture cells as tumorspheres, the patient-derived glioblastoma stem cells were cultured in DMEM/F-12 supplemented with B27, EGF (20 ng/ml), bFGF (20 ng/ml), penicillin-streptomycin (10,000 U/ml) at 37 °C in a humidified atmosphere of 95% air and 5% CO_2_.

### Cell lines, cell culture, and irradiation

U87MG, T98G cell lines were obtained from the Korea Cell Line Bank (KCLB, Seoul, Republic of Korea). The phenotypes of these cell lines have been authenticated by the KCLB. All cells were free of mycoplasma contamination and were authenticated by short tandem repeat profiling within the past 12 months. U87MG-luciferase expressing cells were transferred via a material transfer agreement from Severance Hospital (Yonsei University, Seoul, Republic of Korea). The cells were grown in MEM supplemented with 10% FBS, penicillin (100 U/ml), and streptomycin (100 mg/ml) at 37 °C in a humidified atmosphere of 95% air and 5% CO_2_. The cells were exposed to a single dose of X-ray using an X-ray generator M-150WE (Softex, Tokyo, Japan) at a dose rate of 0.38 Gy/min.

### Animal care protocol and orthotopic xenograft mouse model

Six-week-old male BALB/c athymic nude mice (Orient Bio, Seongnam, Republic of Korea) were used for generating xenograft mouse model following the previous study [18]. All experiments were performed in accordance with the provisions of the NIH Guide for the Care and Use of Laboratory Animals. The mice were housed individually or in groups of up to five in sterile cages, and were maintained in animal care facilities in a temperature regulated room (23 ± 1 °C) with a 12 h light–dark cycle. All animals were fed water and standard mouse chow ad libitum. U87MG-luciferase expressing cells were harvested through trypsinization and suspended at a density of 1 × 10^5^ cells per μl in serum-free media. Then, 5 × 10^5^ cells were injected into the mice brains using stereotactic surgery. 18 days after the injection date, the mice brains were irradiated with 2 Gy daily for five days at a dose rate of 600 MU/min using a TrueBeam STx (Varian Medical Systems, Palo Alto, CA, USA). Xenograft growth was monitored by bioluminescent imaging using VISQUE Invivo Smart LF (Vieworks, Anyang, Republic of Korea). Mice were sacrificed upon manifestation of neurological symptoms.

### Immunofluorescence and live-cell imaging

Cells were fixed in 4% paraformaldehyde at room temperature for 20 min and permeabilized with 0.5% Triton X-100 for 10 min. Subsequently, cells were rinsed three times with PBS, and blocked in blocking buffer (0.1% BSA in PBS) for 30 min. Cells were incubated overnight with the specific primary antibodies at 4 °C, and washed three times with PBS. After being incubated with DyLight 488- or 594-conjugated secondary antibodies (Thermo Scientific), cells were mounted with Fluoroshield Mounting Medium with DAPI (Abcam). Fluorescent images were visualized using a Leica DMi 8 fluorescence microscope (Leica, Wetzlar, Germany). For live cell imaging, cells were grown on glass bottom dishes and treated with CLIP3 siRNA or IR. Intracellular GFP-tagged GLUT3 dynamics in live cells were imaged in an environmentally controlled chamber at 37 °C for the indicated times using LSM 800 confocal microscope (ZEISS, Oberkochen, Germany). The images were analyzed with ZEN software (ZEISS). For analysis of all microscopy images, raw image data were used.

### Total RNA isolation and qRT-PCR

For mRNA expression assessment, qRT-PCR was performed following the previous study [31]. Briefly, RNA was isolated with Trizol following the manufacturers’ instructions and real-time qRT-PCR was performed using an Applied Biosystems StepOne Real-Time PCR System (Applied Biosystems, Foster City, CA, USA). It was performed for 40 cycles of 95 °C for 15 s and 60 °C for 1 min followed by thermal denaturation. The expression of each gene relative to GAPDH mRNA was determined using the 2^-∆∆Ct^ method. The sequences of the primers used are listed in Supplementary Table S2. Each sample was assessed by triplication.

### Western blotting and immunoprecipitation (IP)

The protein expression was validated as previously described [32]. Briefly, whole cell lysates (WCL) were prepared using radioimmunoprecipitation assay (RIPA) lysis buffer (50 mM Tris, pH 7.4, 150 mM NaCl, 1% Triton X-100, 25 mM NaF, 1 mM dithiothreitol, and 20 mM ethylene glycol tetraacetic acid supplemented with protease inhibitors) and the protein concentrations were determined using a BioRad protein assay kit (BioRad Laboratories, Hercules, CA, USA). Protein samples were subjected to SDS-PAGE, transferred to a nitrocellulose membrane and then blocked with 5% bovine serum albumin in tris-buffered saline with Tween 20 (10 mM Tris, 100 mM NaCl, and 0.1% Tween 20). The membranes then were probed using the specific primary antibodies and peroxidase-conjugated secondary antibodies from Santa Cruz Biotechnology. For all western immunoblot experiments, blots were imaged using an ECL detection system (Roche Applied Science, Indianapolis, IN, USA) with iBright FL1000 Imaging System from Thermo Fisher Scientific. For IP studies, we prepared lysates for protein samples obtained from treatment of non-denaturing buffer (50 mM Tris, pH 8.0, 150 mM NaCl, 1% NP-40, 50 mM NaF, 100 μM Na_3_VO_4_, 50 μM PMSF, 2 μg/ml Aprotinin, 1 μg/ml Leupeptin) for 30 min at 4 °C, or treatment of denaturing buffer (50 mM Tris, pH 8.0, 150 mM NaCl, 1% NP-40, 0.1% SDS, 0.5% sodium deoxycholate, 50 mM NaF, 100 μM Na_3_VO_4_, 50 μM PMSF, 2 μg/ml Aprotinin, 1 μg/ml Leupeptin) for 5 min at 95 °C. The lysates for protein samples were immunoprecipitated overnight with the specific primary antibodies and protein A/G agarose beads (Santa Cruz Biotechnology). After washing with the lysis buffer, immunoprecipitates were then boiled in 2 × SDS sample buffer for 10 min, followed by centrifugation. They were detected by Western blot analysis.

### Luciferase reporter gene assay

Genomic region harboring CLIP3 promoter (300 bp upstream of the transcription start site of the gene) was cloned into pGL3-NFAT luciferase vector digested by MluI and HindIII. The sequences of the wild-type or mutant CLIP3 promoter are listed in Supplementary Table S3. A luciferase assay was performed as previously reported [33]. Briefly, luciferase activity was measured using Luciferase Assay System from Promega (Madison, WI, USA). Cells were seeded in 60 mm culture dishes 1 day before transfection. At 48 h after transfection, media was removed and the dishes were washed by PBS. 400 μl of Cell Culture Lysis Reagent was added to the dishes directly and transferred to new tubes. After brief centrifugation, 20 μl of cell lysate was mixed with 100 μl of Luciferase Assay Reagent. Luminescence was measured using a Glomax multi detection system (Promega).

### Flow cytometry

The expression of the molecular marker CD133 in the various cell cultures was detected using an anti-CD133-PE antibody. Cells were gently disaggregated to single-cell suspensions by trypsin, and stained with CD133-PE for 30 min in the dark at 4 °C. The stained cells were then detected using a FACSVerse flow cytometer (BD Biosciences, San Jose, CA, USA).

### In vitro limiting dilution neurosphere formation assay

For in vitro limiting dilution assays, decreasing numbers of cells per well (500, 200, 100, 50, 20, and 10) were plated into 96-well plates. The presence and number of neurospheres in each well were recorded 10 days after plating. Extreme limiting dilution analysis was performed using software available at http://bioinf.wehi.edu.au/software/elda [34].

### Measurement of OCR and ECAR

Oxygen consumption rate (OCR) and extracellular acidification rate (ECAR) were measured by Seahorse XFp Analyzer (Agilent Technologies, Santa Clara, CA, USA) with 80–90% confluent cells following the previous study [18]. Briefly, on the day following cell seeding and treatment of siRNA or glimepiride, cells were equilibrated for 1 h in a non-CO_2_ incubator. For the OCR assay, the media were changed to the XF assay media (Agilent Technologies). Injection port A on the sensor cartridge was loaded with oligomycin (2 μM), port B was loaded with FCCP (1 μM), and port C was loaded with rotenone/antimycin A (1 μM each). For the ECAR assay, the media were changed to the XF assay media (Agilent Technologies) without glucose. The injection port A was loaded with glucose (10 mM), port B was loaded with oligomycin (2 μM), and port C was loaded with 2-Deoxy-D-glucose (100 mM). A minimum of three wells were utilized per condition to calculate OCR and ECAR.

### Metabolic assays

Metabolic assays were performed following the previous study [18]. In brief, glucose uptake, lactate production, and the levels of G3P, serine, fumarate, malate, and ATP were measured using assay kits from BioVision (San Francisco, CA, USA). NADP^+^/NADPH ratio and the levels of citrate and succinate were determined using assay kits from Abcam.

### Cell viability assay and colony-forming assay

For cell viability assay, cells were seeded at 10,000 cells per well in 96-well plates 1 day before the addition of glimepiride and/or IR for 48 h. Cell viability was determined using CellTiter-Glo® Luminescent Viability Assay kit (Promega). Colony-forming assay was performed following the previous study [35]. Briefly, the cells were seeded at a density of 600 cells in 35-mm culture dishes. After 24 h, the cells were treated with glimepiride and/or IR. 14 days after seeding, the cells were fixed with 10% methanol and 10% acetic acid, which were then stained with 1% crystal violet. Colonies containing more than 50 cells were identified using densitometry software and scored as survivors.

### Hematoxylin and eosin (H&E) staining and immunohistochemistry (IHC)

H&E staining and IHC were performed as previously described [36]. The brain samples were embedded in paraffin blocks, and the sections were prepared by HistoCore AutoCut (Leica, Deerfield, IL, USA). Next, the sections were cut into 4 μm sections and stained with H&E, following standard procedures. For IHC, sections were treated with 3% hydrogen peroxide/methanol and then with 0.25% pepsin to retrieve antigens. Next, samples were incubated in blocking solution (Dako, Carpinteria, CA, USA), after which they were incubated at 4 °C overnight with the specific primary antibodies diluted in the antibody diluent (Dako). The sections were subsequently washed with tris buffered saline with 0.1% Tween 20 and then incubated with polymer-horseradish peroxidase-conjugated secondary antibody (Dako). A 3,3′-diaminobenzidine substrate chromogen system (Dako) was utilized to detect antibody binding. Stained sections were observed under an Olympus IX71 inverted microscope (Olympus Optical, Tokyo, Japan).

### Statistical analysis

All numerical data are presented as the means ± standard error of the mean from at least three independent experiments. For quantifications, two-tail unpaired Student’s *t*-test was used for comparing two experimental groups, and one-way ANOVA was applied when needed to compare three or more experimental groups. Log-rank (Mantel-Cox) test was used for statistical analysis of survival. The Prism 9 software (GraphPad Software, San Diego, CA, USA) was used for all statistical analyses. A *p*-value < 0.05 was considered to be statistically significant.

## Results

### GBM cells acquire radioresistance by CLIP3 downregulation

**Fig. 1:**
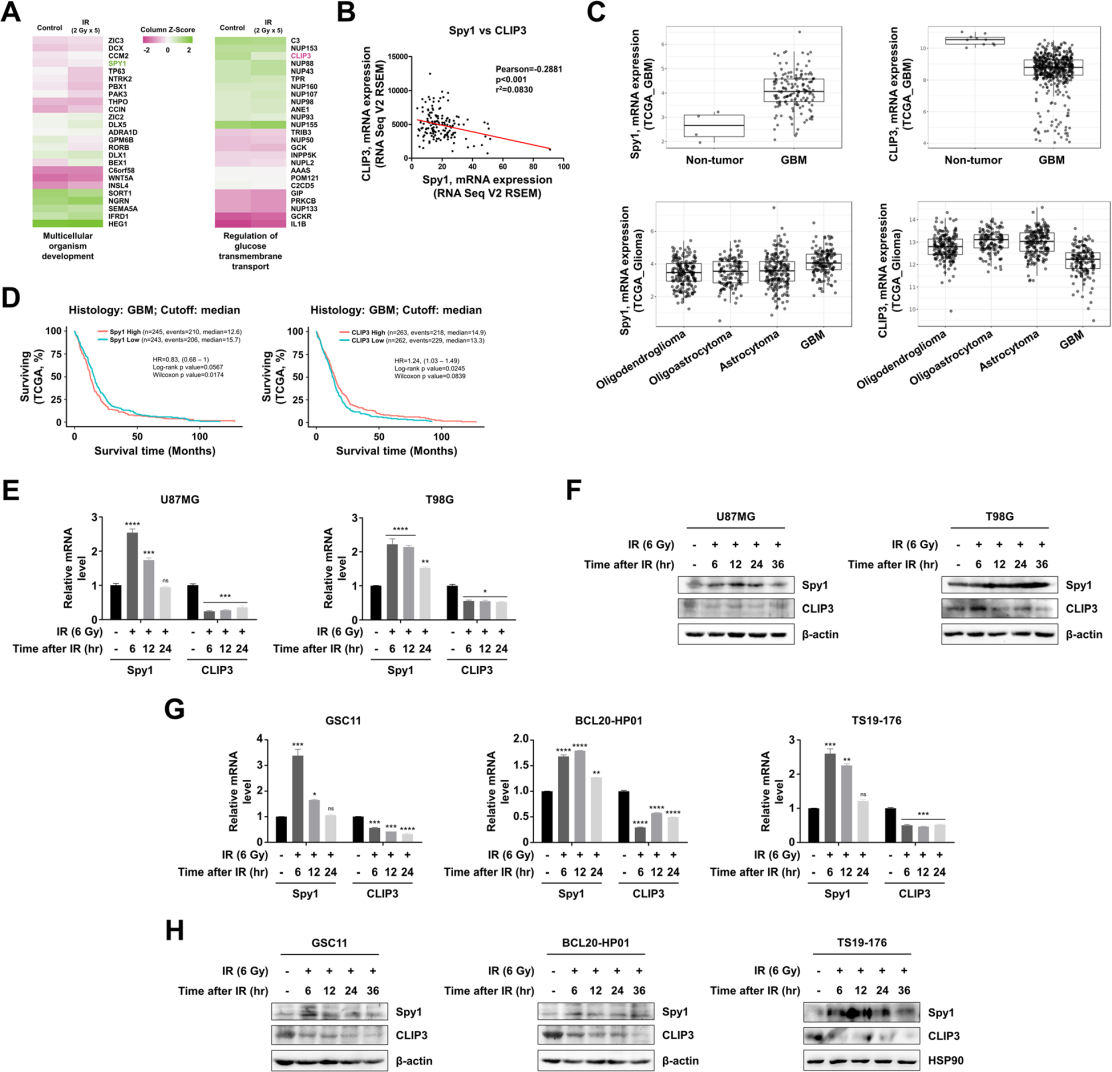
GBM cells acquire radioresistance by CLIP3 downregulation. **A** cDNA microarray analysis was performed in orthotopic xenograft GBM tumor sections from control and IR (2 Gy × 5) groups. **B**, **C** The gene expression profiles and clinical data were collected from The Cancer Genome Atlas (TCGA) database available through Gliovis [37]. **B** Correlation of mRNA expression levels between Spy1 and CLIP3 in 152 GBM patients (RNA Seq V2 RSEM). **C** mRNA expression levels of Spy1 and CLIP3 were investigated in 10 non-tumor tissues and 528 GBM tissues (upper panel), and in 515 low-grade glioma and 152 GBM tissues (lower panel). **D** Survival rate calculated by the Kaplan-Meier survival curve in GBM patients separated according to median expression level of Spy1 and CLIP3. **E** mRNA levels of Spy1 and CLIP3 were analyzed by real-time qRT-PCR at 6, 12, and 24 h after IR (6 Gy) in U87MG and T98G cells. **F** Protein levels of Spy1, CLIP3, and β-actin were analyzed by western blot at 6, 12, 24, and 36 h after IR (6 Gy) in U87MG and T98G cells. **G** mRNA levels of Spy1 and CLIP3 were analyzed by real-time qRT-PCR at 6, 12, and 24 h after IR (6 Gy) in GSC11, BCL20-HP01, and TS19–176 cells. **H** Protein levels of Spy1, CLIP3, β-actin, and HSP90 were analyzed by western blot at 6, 12, 24, and 36 h after IR (6 Gy) in GSC11, BCL20-HP01, and TS19–176 cells. Statistical analysis was performed with one-way ANOVA plus a Tukey’s multiple comparisons test. *p < 0.05, **p < 0.01, ***p < 0.001, ****p < 0.0001

We previously established a GBM orthotopic xenograft mouse model in which we analyzed gene expression of cells that survived cranial radiation (2 Gy/day for 5 days) (GEO accession number: GSE117126) [18]. Of the various genes analyzed, genes related to stemness or glucose metabolism were the most highly upregulated in surviving cells relative to control cells, and were hence considered the most relevant to conferring radioresistance and tumor recurrence. Within the categories ‘multicellular organism development’ and ‘Regulation of glucose transmembrane transport’ ontologies, we decided to focus on Spy1 and CLIP3 based on their inverse expression pattern and the published literature (Fig. 1A); Spy1 expression increased in irradiated GBM cells, while CLIP3 expression decreased. We hypothesized that as Spy1 and CLIP3 altered expression was induced by radiation and conferred radioresistance, these two proteins would regulate mechanism that could be therapeutically targeted. According to the TCGA data of the 152 GBM patients, Spy1 and CLIP3 gene expression levels were negatively correlated (Fig. 1B). Likewise, Spy1 expression was greatly upregulated in GBM patients in comparison to normal brain tissues or low-grade glioma tissues, while CLIP3 expression was highly downregulated in GBM tissues (Fig. 1C). In addition, both Spy1 and CLIP3 functioned as biomarkers; Spy1 expression informed poor prognosis, whereas CLIP3 expression correlated positively with patient outcomes (Fig. 1D and S1A).

To examine gene expression profiles of cells that survive IR in vitro, we measured Spy1 and CLIP3 expression levels in two GBM cell lines (U87MG and T98G) in time course experiments upon IR (6 Gy) (Fig. 1E and F). Spy1 expression increased shortly after IR (6, 12 h), while CLIP3 expression was constantly decreased at both mRNA and protein levels. In addition, IR in three patient-derived glioblastoma stem cell lines (GSC11, BCL20-HP01, and TS19–176) resulted in similar Spy1 and CLIP3 expression level changes (Fig. 1G and H). Taken together, we reasoned that IR exposure-induced upregulation of Spy1 and downregulation of CLIP3 contributed to the radioresistance acquisition of surviving GBM cells.

### Spy1 negatively regulates the transcriptional activity of CLIP3 by CDK2/NRF1 signaling

**Fig. 2:**
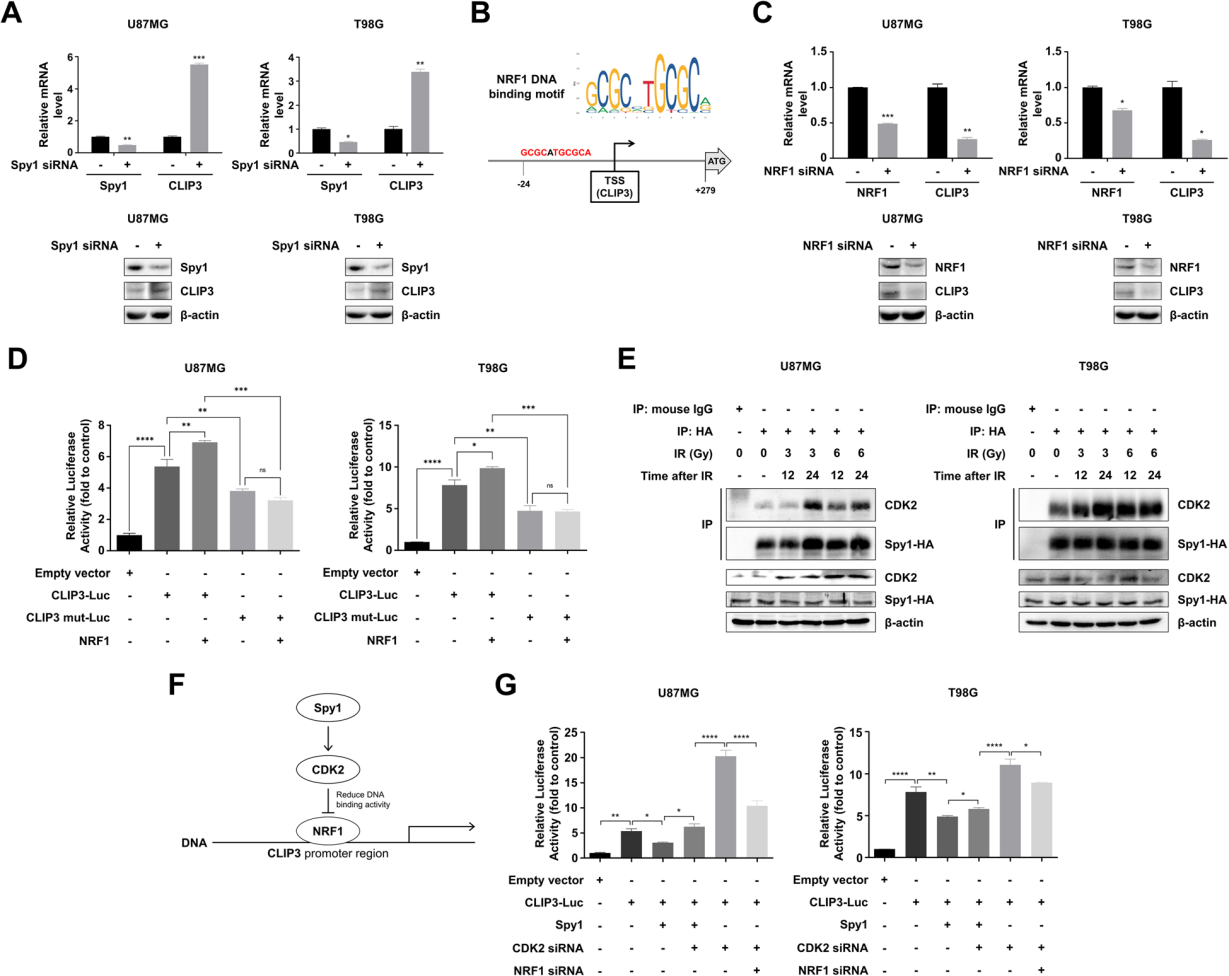
Spy1 negatively regulates the transcriptional activity of CLIP3 by CDK2/NRF1 signaling. **A** mRNA and protein levels of Spy1 and CLIP3 were analyzed by real-time qRT-PCR and Western blot, respectively, upon Spy1 siRNA treatment in U87MG and T98G cells. **B** A possible NRF1 binding site on the CLIP3 promoter was predicted using GeneCards database (https://www.genecards.org/). NRF1 was expected to bind − 24 to − 14 (GCGCATGCGCA) from the transcription start site (TSS) of CLIP3. **C** mRNA and protein levels of NRF1 and CLIP3 were analyzed by real-time qRT-PCR and Western blot, respectively, upon NRF1 siRNA treatment in U87MG and T98G cells. **D** Luciferase activity was measured upon transfection of pGL3-NFAT-luc plasmids, including wild-type (CLIP3-Luc) or mutant CLIP3 (CLIP3 mut-Luc) promoter linked to luciferase gene, in the absence or presence of transfection of NRF1 gene in U87MG and T98G cells. **E** Western blot with CDK2 or HA antibodies after immunoprecipitation of Spy1 from U87MG and T98G whole cell lysates at 12 and 24 h after IR (3 or 6 Gy) using a HA antibody. Protein levels of CDK2, Spy1, and β-actin in the whole cell lysates were analyzed at 12 and 24 h after IR (3 or 6 Gy) by western blot. **F** A schematic diagram illustrates that Spy1 negatively regulates CLIP3 through CDK2/NRF1 signaling. **G** Luciferase activity was measured upon transfection of the pGL3-NFAT-luc plasmids with Spy1 gene, CDK2 siRNA, or NRF1 siRNA treatment in U87MG and T98G cells. Statistical analysis was performed with Student’s *t*-test for (**A**) and (**C**), and one-way ANOVA plus a Tukey’s multiple comparisons test for (**D**) and (**G**). *p < 0.05, **p < 0.01, ****p < 0.0001

To investigate whether the negative correlation between Spy1 and CLIP3 represented causation, we experimentally tested whether a transient increase of Spy1 after IR reduces CLIP3 transcription levels. As shown in Fig. 2A, knockdown of Spy1 increased CLIP3 mRNA and protein level in GBM cell lines. When we used GeneCards database to identify potential upstream regulator of CLIP3 transcription, we found that nuclear respiratory factor 1 (NRF1) was top-ranked (Fig. 2B). Accordingly, knockdown of NRF1 dramatically decreased CLIP3 mRNA and protein level in GBM cell lines, implying that NRF1 is a transcriptional activator of CLIP3 (Fig. 2C). To verify that NRF1 directly binds to the promoter region of CLIP3, we transfected pGL3-NFAT-luc plasmids (wild-type or mutant CLIP3 promoter linked to luciferase gene) in the absence or presence of NRF1 gene transfection, and measured luciferase activity in GBM cell lines (Fig. 2D). Indeed, we found that luciferase activity was increased with the wild-type CLIP3 promoter upon transfection of NRF1, while it hardly changed with the mutant CLIP3 promoter. In addition, the luciferase activity with the CLIP3 mut-Luc promoter significantly decreased compared to with the CLIP3 wt-Luc promoter either in the absence or presence of NRF1, reflecting that NRF1 bound to its consensus sequence (GCGCATGCGCA) within CLIP3 promoter region and played a role as a transcriptional activator. Interestingly, a recent study revealed that CDK2 activation decreased the DNA binding activity of NRF1, which is known to be induced by Spy1 [38]. Because CDK2 contributes to radioresistance by activating S phase, we next examined the binding affinity of Spy1 and CDK2 after IR in GBM cell lines (Fig. 2E) [39]. In immunoprecipitation experiments, we found that IR caused CDK2 to bind to HA-tagged Spy1, which implied CDK2 activation, in a time- and dose-dependent manner. Therefore, we hypothesized that Spy1 could negatively regulate the transcriptional level of CLIP3 by releasing NRF1 from the CLIP3 promoter region (Fig. 2F). To test this hypothesis, we transfected GBM cell lines with the CLIP3 promoter, and measured luciferase activity upon Spy1 overexpression with or without CDK2 knockdown (Fig. 2G). Our data showed that Spy1 overexpression increased luciferase activity, but it was reduced with the CDK2 knockdown. We then measured the activity upon knockdown of CDK2 only, or both CDK2 and NRF1 (Fig. 2G). CDK2 knockdown significantly increased luciferase activity, but an additional knockdown of NRF1 decreased the activity. Collectively, these data demonstrated that downregulation of CLIP3 expression in GBM cells is mediated by IR-induced Spy1 expression.

### Acquisition of stemness is mediated by Spy1-CLIP3 axis in GBM cells

**Fig. 3:**
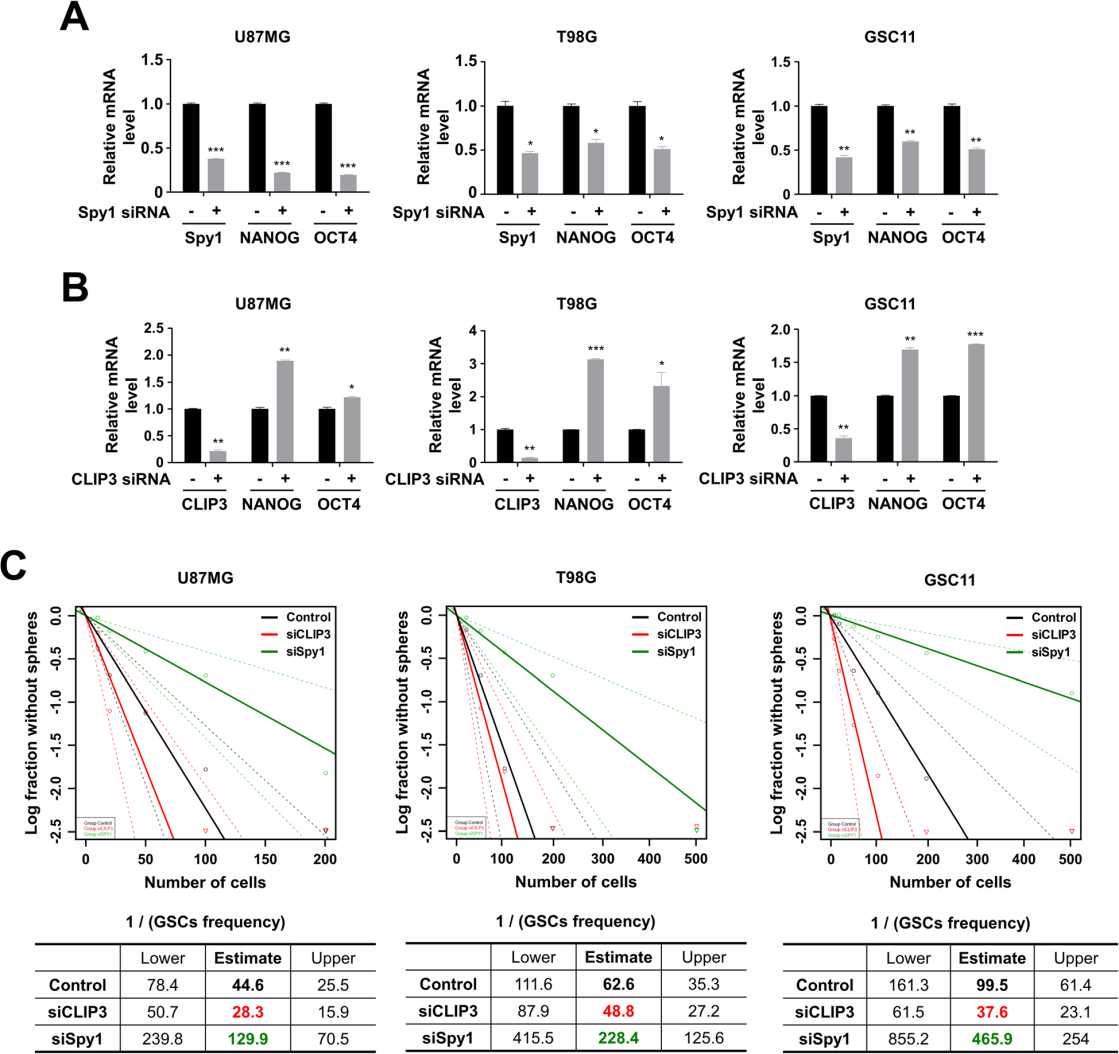
Acquisition of stemness is mediated by Spy1-CLIP3 axis in GBM cells. **A** mRNA levels of Spy1, NANOG, and OCT4 were analyzed by real-time qRT-PCR upon Spy1 siRNA treatment in U87MG, T98G, and GSC11 cells. **B** mRNA levels of CLIP3, NANOG, and OCT4 were analyzed by real-time qRT-PCR upon CLIP3 siRNA treatment in U87MG, T98G, and GSC11 cells. **C** In vitro limiting dilution assays of U87MG, T98G, and GSC11 cells treated with Spy1 or CLIP3 siRNA. The frequency of GSCs was calculated by extreme limiting dilution assay (ELDA) analysis. *p < 0.05, **p < 0.01, ***p < 0.001 with unpaired *t*-test

Because Spy1 appears to primarily maintain GSC self-renewal as the mechanism driving GBM cell radioresistance [10], we next investigated how CLIP3 downregulation impacts on stemness properties of GBM cells. After exposure to IR or CLIP3 siRNA, flow cytometry sorted cells were significant enriched for cells expressing CD133 (Prominin1), a representative GSC surface marker, (Fig. S2A). Similarly, Spy1 knockdown suppressed the expression of canonical stem cell transcription factors NANOG and OCT4 in both GBM cell lines and patient-derived GSC11 glioblastoma stem cells (Fig. 3A). On the other hand, CLIP3 knockdown significantly increased NANOG and OCT4 expression (Fig. 3B). The degree of GSC stemness is reflected in capacity to form tumor spheres in GSC culture. Indeed, in limiting dilution assays using U87MG, T98G, and GSC11, we found that the frequency of GSCs capable of forming tumor spheres was decreased by Spy1 knockdown but increased by CLIP3 knockdown (Fig. 3C). Taken together, CLIP3 downregulation enhances GBM stemness without any further activation of Spy1.

### CLIP3 controls plasma membrane translocation of GLUT3 in GBM cells

**Fig. 4:**
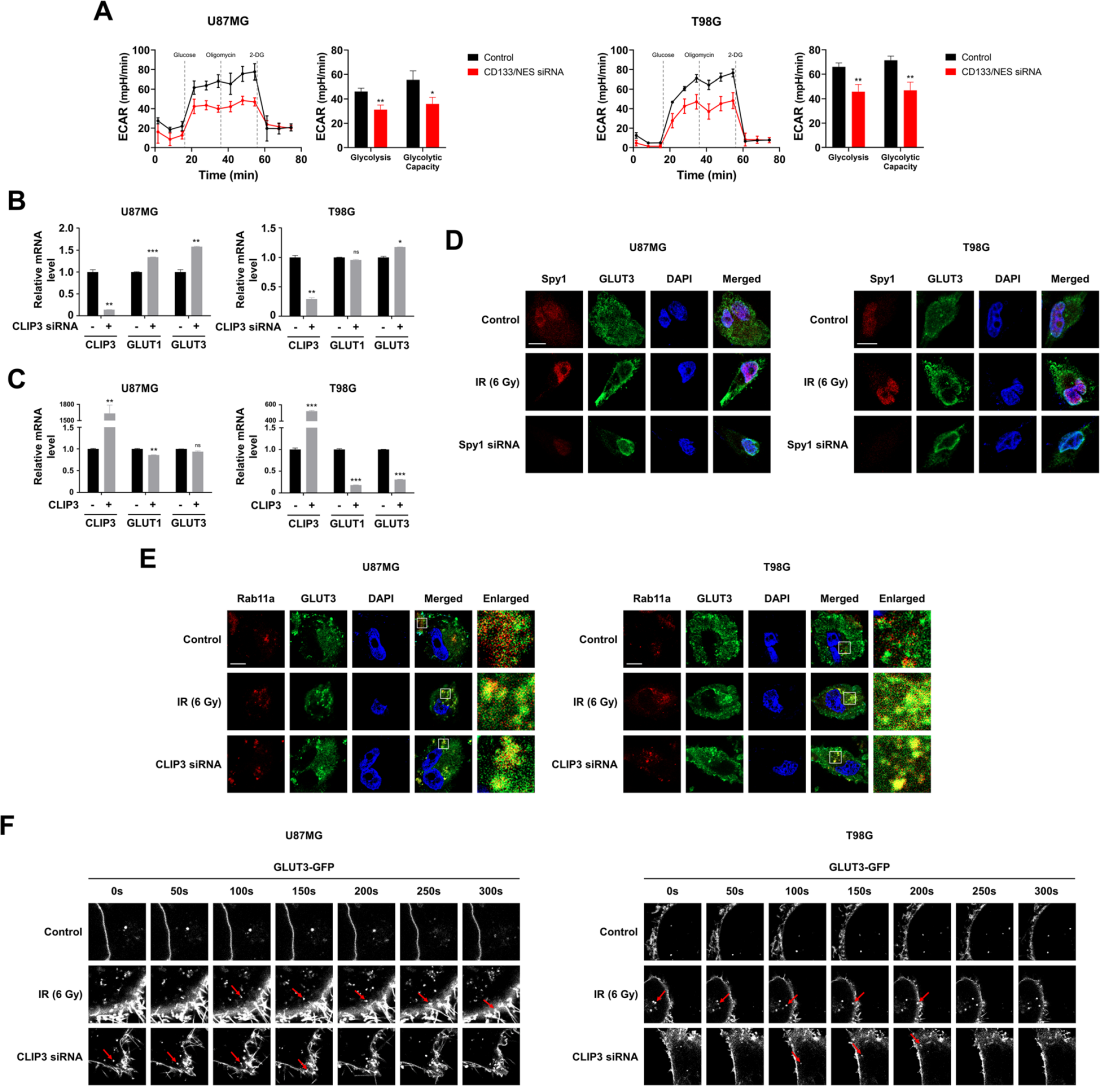
CLIP3 controls plasma membrane translocation of GLUT3 in GBM cells. **A** ECAR was measured by Seahorse analyzer upon transfection of both CD133 and NES siRNA in U87MG and T98G cells. Bar graphs depict glycolysis (measured by the generation of lactate upon glucose addition) and glycolytic capacity (the maximum capacity of lactate generation upon inhibition of oxidative phosphorylation). 2-DG, 2-Deoxy-D-glucose. **B** mRNA levels of CLIP3, GLUT1, and GLUT3 were analyzed by real-time qRT-PCR upon CLIP3 siRNA treatment in U87MG and T98G cells. **C** mRNA levels of CLIP3, GLUT1, and GLUT3 were analyzed by real-time qRT-PCR upon transfection of CLIP3 gene in U87MG and T98G cells. **D** Representative immunofluorescence staining for Spy1 (red), GLUT3 (green), and merged images (with DAPI, blue) on Control, IR (6 Gy), or Spy1 siRNA treatment in U87MG and T98G cells. Scale bars, 10 μm. **E** Representative immunofluorescence images for Rab11a-OFP (red), GLUT3-GFP (green), and merged images on Control, IR (6 Gy), or CLIP3 siRNA treatment in U87MG and T98G cells. Scale bars, 10 μm. **F** Confocal live-cell images of U87MG and T98G cells stably expressing GLUT3-GFP after treatment of IR (6 Gy) or CLIP3 siRNA for the indicated times. Arrows indicate GLUT3-GFP-containing vesicular structures migrating toward the plasma membrane. Scale bars, 5 μm. *p < 0.05, **p < 0.01, ***p < 0.001 with unpaired *t*-test

Given that CSCs have higher glycolytic activity than other cancer cells, and CLIP3 is related to regulation of glucose transmembrane transport (Fig. 1A), we next examined these functional relationships by measuring the extracellular acidification rate (ECAR) with Seahorse XFp analyzer. As shown in Fig. 4A, double-knockdown of CD133 and NESTIN (NES) significantly suppressed glycolytic rate and capacity in U87MG and T98G cells, reflecting that glycolytic activity of GBM is highly related to GSC enrichment. Next, we assumed that downregulation of CLIP3 after IR exposure might elevate glycolytic activity by regulating GLUTs, which are critical for GBM cell activity. In U87MG cells, levels of both GLUT1 and GLUT3, the major GLUTs in GBM, were increased upon CLIP3 knockdown, while in T98G cells, only the GLUT3 level was slightly increased (Fig. 4B). On the other hand, CLIP3 overexpression barely decreased GLUT mRNA expression in U87MG cells while levels of both GLUTs were significantly decreased by CLIP3 transfection in the T98G cells (Fig. 4C), showing that GLUT transcription was not completely regulated by CLIP3 expression. As the cellular location of the GLUTs is known to be impacted by CLIP3, we next used immunocytochemistry after IR exposure to investigate cellular localization of GLUTs [12, 13]. As shown in Fig. 4D, IR considerably increased nuclear expression of Spy1, and accordingly, membrane translocation of GLUT3, but not GLUT1 in GBM cell lines. On the other hand, Spy1 knockdown restricted GLUT3 membrane localization. In a previous study, GLUT3 translocation was mediated by the evasion of autophagic degradation of Rab11a-positive endosome, and we therefore next examined whether IR and CLIP3 knockdown could locate GLUT3 on the Rab11a-positive endosome. Immunocytochemistry analysis shows that colocalization of Rab11a and GLUT3 was increased after IR and CLIP3 knockdown in GBM cell lines (Fig. 4E). Furthermore, live cell imaging of GBM cell lines expressing GLUT3-GFP revealed that both IR and CLIP3 knockdown induced GLUT3 cell surface trafficking (Fig. 4F and Supplementary Videos S1, S2, S3, S4, S5 and S6). In summary, IR triggers downregulation of CLIP3 which increases glycolytic activity by promoting Rab11a-dependent membrane translocation and recycling of GLUT3, the key GLUT in GSCs.

### Spy1-CLIP3 axis contributes to metabolic shift toward glycolysis in GBM cells

**Fig. 5:**
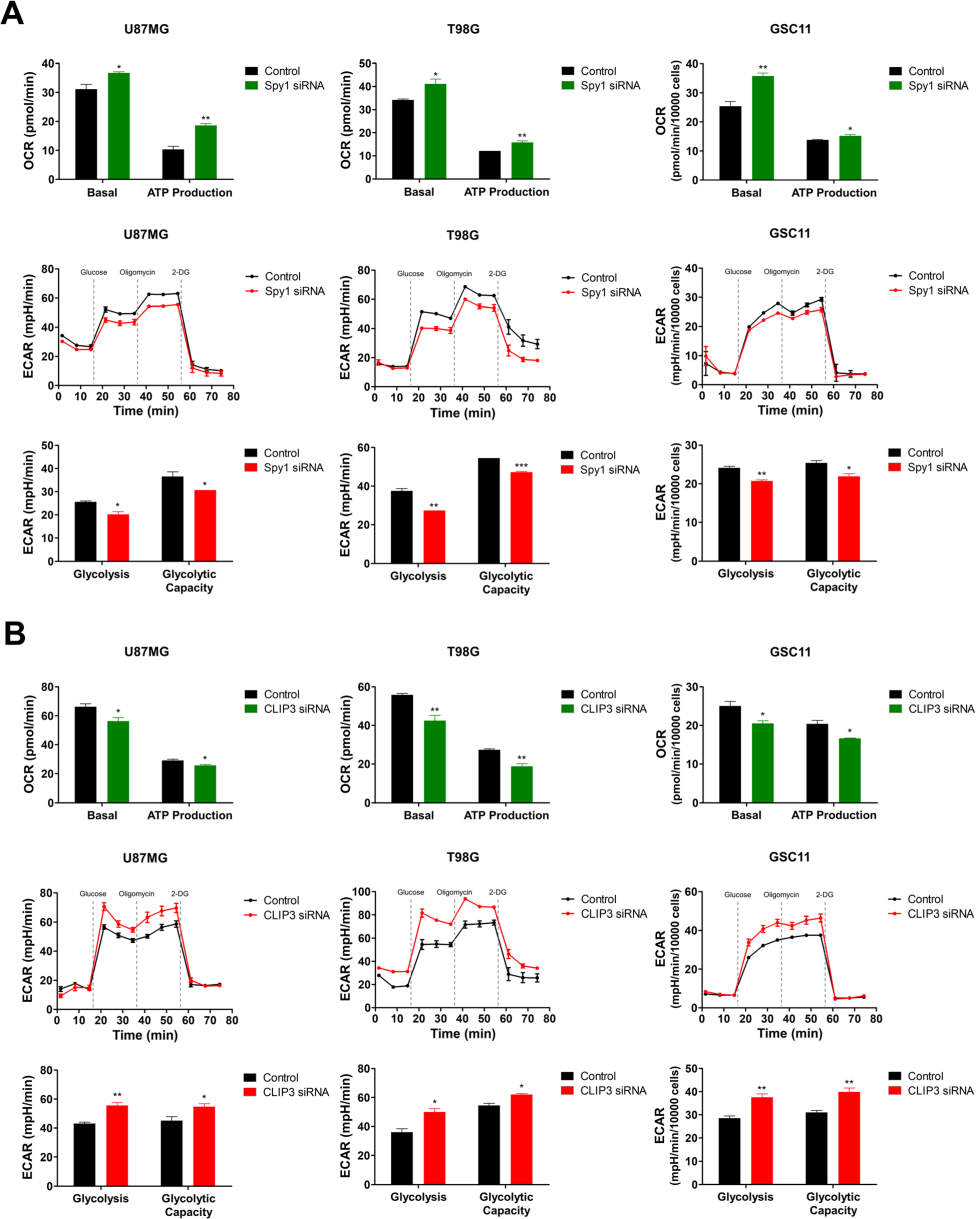
Spy1-CLIP3 axis contributes to metabolic shift toward glycolysis in GBM cells. **A**, **B** OCR and ECAR were measured by Seahorse analyzer in U87MG, T98G, and GSC11 cells. Basal OCR indicates the basal level of oxygen consumption and ATP production indicates the decrease in oxygen consumption rate upon injection of oligomycin, which represents the portion of basal respiration that was being used to drive ATP production. Glycolysis indicates the generation of lactate upon glucose addition and glycolytic capacity indicates the maximum capacity of lactate generation upon inhibition of oxidative phosphorylation. OCR and ECAR upon transfection of Spy1 siRNA (**A**) or CLIP3 siRNA (**B**). *p < 0.05, **p < 0.01, ***p < 0.001 with unpaired *t*-test

To determine whether Spy1 and CLIP3 directly control glycolytic activity, we tested the oxygen consumption rate (OCR) and ECAR in the GBM cell lines (U87MG and T98G) and patient-derived glioblastoma stem cells (GSC11, BCL20-HP01, and BCL20-HP02). After Spy1 knockdown, both basal OCR and ATP production were increased, whereas glycolytic rate and capacity were downregulated (Fig. 5A and S3A). Conversely, CLIP3 knockdown decreased basal OCR and ATP production, and increased glycolytic rate and capacity (Fig. 5B and S3B). We then measured major intermediates of glucose metabolism (Fig. S4A) in U87MG cells after IR exposure, Spy1 overexpression, CLIP3 knockdown, IR with Spy1 knockdown, or IR with CLIP3 overexpression (Fig. S4B-S4E). As shown in Fig. S4B, both glucose uptake and lactate production were elevated after IR, Spy1 overexpression, or CLIP3 knockdown, but were restored by IR with Spy1 knockdown or IR with CLIP3 overexpression. Similarly, in Fig. S4C, glycerol-3-phosphate and serine levels were also increased in the elevated groups, but the NADP^+^/NADPH ratio was reduced, which would mean that glycolysis-related pathways and the pentose phosphate pathway were also activated by IR, Spy1, or CLIP3 knockdown. We also found that production of citric acid cycle intermediates was decreased after IR, Spy1 overexpression, or CLIP3 knockdown, and rescued by IR with Spy1 knockdown or IR with CLIP3 overexpression (Fig. S4D). In Fig. S4E, we forced the cells to undergo either glycolysis or oxidative phosphorylation. ATP levels were not significantly changed in normoxic conditions across all groups, but in hypoxic conditions, ATP levels were increased after IR in cells with Spy1 overexpression or CLIP3 knockdown, but were almost restored to control levels after IR with Spy1 knockdown or CLIP3 overexpression. Conversely, upon glucose deprivation, we found that cell numbers were significantly decreased after IR in cells with Spy1 overexpression or CLIP3 knockdown due to glycolytic dependency, but cell numbers significantly recovered after IR in cells with Spy1 knockdown or CLIP3 overexpression. Taken together, our data are consistent with an IR response in which GBM cells switch glucose metabolism towards glycolysis through Spy1 activation and CLIP3 inhibition.

### Glimepiride disrupts GSC maintenance and glycolytic activity by CLIP3 activation

**Fig. 6:**
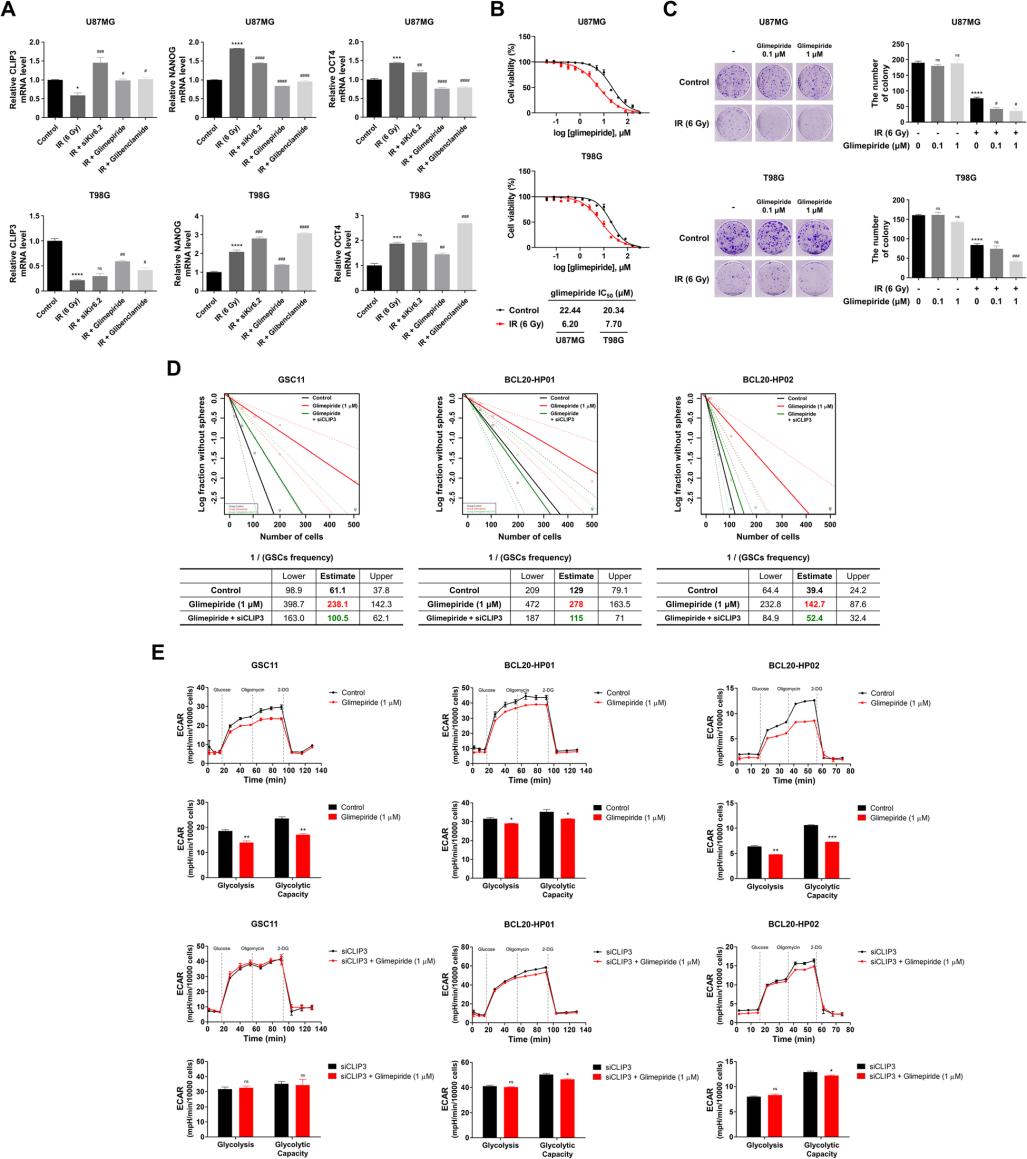
Glimepiride disrupts GSC maintenance and glycolytic activity by CLIP3 activation. **A** mRNA levels of CLIP3, NANOG, and OCT4 were analyzed by real-time qRT-PCR upon treatment of IR (6 Gy), IR with Kir6.2 siRNA, IR with glimepiride (1 μM), or IR with glibenclamide (1 μM) in U87MG and T98G cells. **B** IC_50_ of glimepiride with or without IR in U87MG and T98G cells was measured by CellTiter-Glo® Luminescent Cell Viability Assay. Cells were treated with increasing concentrations of glimepiride and/or IR (6 Gy) for 48 h. **C** Colony-forming ability was evaluated using colony-forming assay after treatment of glimepiride (0.1 and 1 μM) with or without IR (6 Gy) (data represent mean of *n* = 3 dishes). **D** In vitro limiting dilution assays of GSC11, BCL20-HP01, and BCL20-HP02 cells treated with glimepiride (1 μM) or glimepiride with CLIP3 siRNA. The frequency of GSCs was calculated by extreme limiting dilution assay (ELDA) analysis. **E** ECAR was measured by Seahorse analyzer upon treatment of glimepiride (1 μM) (upper panel), and treatment of CLIP3 siRNA with or without glimepiride (lower panel) in GSC11, BCL20-HP01, and BCL20-HP02 cells. Statistical analysis was performed with one-way ANOVA plus a Tukey’s multiple comparisons test for (**A**) and (**C**), and Student’s *t*-test for (**E**). *p < 0.05, **p < 0.01, ***p < 0.001, ****p < 0.0001 (compared to control). ^#^*p* < 0.05, ^##^*p* < 0.01, ^###^*p* < 0.001, ^####^*p* < 0.0001 (compared to IR)

We next investigated drugs that could regulate the Spy1-CLIP3 axis. Because the expression of CLIP3, but not Spy1, constantly changed after IR, we focused on CLIP3 activating drugs that might have a capacity for increasing GBM cell radiosensitivity. To this end, we interrogated The Connectivity Map (CMap), a database of drug-induced gene expression profiles, to discover candidates among existing drugs [40]. Of the many candidates, glimepiride, which was FDA-approved in 1995 for type 2 diabetes mellitus, significantly increased transcriptional levels of CLIP3 [41]. Because glimepiride is known to bind to and close ATP-sensitive K^+^ (K_ATP_) channels, including the inward-rectifier potassium channel 6.2 (Kir6.2), we investigated not only the effect of glimepiride as a radiosensitizer but also that of Kir6.2 knockdown or glibenclamide belonging to second-generation sulfonylurea such as glimepiride [42]. First, we verified whether glimepiride (1 μM), glibenclamide (1 μM), or Kir6.2 knockdown with IR exposure could restore CLIP3 transcription and attenuate that of NANOG and OCT4 to reduce radioresistance (Fig. 6A). Of the three different treatments, only glimepiride was able to significantly increase CLIP3 mRNA level and decrease NANOG and OCT4 mRNA levels after IR in GBM cell lines. Similarly, in patient-derived GSC11 glioblastoma stem cells, glimepiride significantly increased CLIP3 expression and reduced NANOG and OCT4 expression without IR exposure due to its high intrinsic radioresistance (Fig. S5A). We next tested the effect of IR on glimepiride sensitivity using CellTiter-Glo® Luminescent Cell Viability Assay (Fig. 6B). IR significantly improved glimepiride sensitivity of both U87MG and T98G cell lines. IR reduced the IC_50_ of glimepiride from 22.44 μM to 6.20 μM in U87MG cells and from 20.34 μM to 7.70 μM in T98G cells. Additionally, we further examined synergistic effects of IR and glimepiride (0.1 and 1 μM) by colony-forming assay. Glimepiride did not reduce colony-forming ability in non-irradiated cells, but with IR, unlike the cell viability assay, which showed little cytotoxicity at 1 μM, colony-forming ability was significantly reduced by 1 μM of glimepiride with IR in U87MG and T98G cells, presumably because glimepiride disrupted GSCs maintenance (Fig. 6C). Furthermore, the combination therapy reduced glucose uptake and lactate production compared with IR only (Fig. S6A and S6B). These data suggest that glimepiride acts as a radiosensitizer by suppressing stemness and glycolysis in GBM cells. To further validate these results, we examined effect of glimepiride on the patient-derived glioblastoma stem cells (GSC11, BCL20-HP01, and BCL20-HP02) (Fig. 6D and E). As shown in Fig. 6D, glimepiride (1 μM) significantly reduced sphere-forming ability, but this effect was diminished with CLIP3 knockdown. Similarly, the glycolytic rate and capacity were markedly decreased upon glimepiride treatment (1 μM), but it was not able to change ECAR without CLIP3 expression in all three GSCs (Fig. 6E). Taken together, our data show that glimepiride can disrupt GSC maintenance and glycolysis, and overcome radioresistance by activating CLIP3.

### Combination of glimepiride with IR improves survival of GBM-bearing mice

**Fig. 7:**
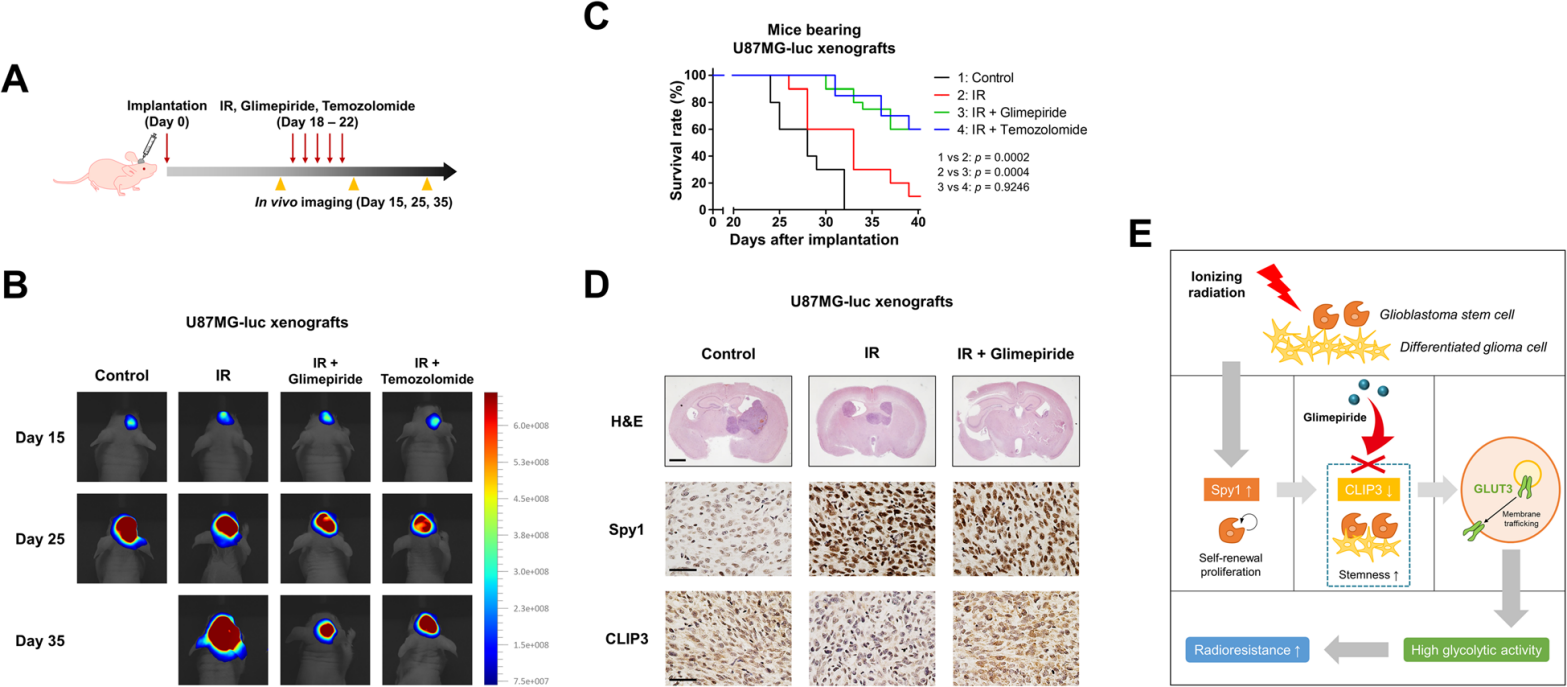
Combination of glimepiride with IR improves survival of GBM-bearing mice. **A** A schematic diagram of control, IR (2 Gy × 5), IR with glimepiride (5 mg/kg, oral), and IR with temozolomide (20 mg/kg, i.p.) treatment in mice bearing U87MG-luciferase xenografts (*n* = 20 mice per group). **B** In vivo bioluminescent images of orthotopic xenografts derived from U87MG-luciferase in mice control, treated with IR, IR with glimepiride, or IR with temozolomide. **C** Survival analysis by Kaplan-Meier curves and log-rank (Mantel-Cox) test of mice bearing U87MG-luciferase xenografts control, treated with IR, IR with glimepiride, or IR with temozolomide. **D** Hematoxylin and eosin (H&E) staining (upper panel) and immunohistochemistry (anti-Spy1 and anti-CLIP3; middle and lower panel) of coronal sections from mice bearing U87MG-luciferase xenografts control, treated with IR, or IR with glimepiride. Scale bars, 2000 μm (upper) or 50 μm (lower). **E** Schematic diagram depicting that CLIP3 activation by glimepiride impairs radioresistance of GSCs

To examine effects of glimepiride in vivo and compare the effects with the first-line drug TMZ, U87MG (MGMT negative) was used instead of T98G (MGMT positive). We implanted U87MG-luciferase expressing cells (U87MG-luc) orthotopically in BALB/c nude mice (Fig. 7A). The mice were treated with IR alone, or IR in combination with glimepiride or TMZ two weeks after the orthotopic xenograft. In vivo bioluminescent imaging showed that IR/glimepiride combination treatment significantly inhibited tumor growth and conferred a marked survival benefit in comparison to untreated control (28 days of median survival) or IR-alone (33 days of median survival), and was even as effective as TMZ combined with IR (Fig. 7B and C). Furthermore, even though glimepiride is a diabetic medicine, the body weight of the mice hardly changed, indicating little side effect (Fig. S7A). To verify the lack of tumor growth and measure protein expression in vivo, we conducted a histological analysis of the brain tissue. Consistent with the in vitro analysis, expression and nuclear localization of Spy1 were highly increased by IR, and IR/glimepiride combination therapy strongly suppressed tumor growth, but also activated CLIP3 expression, resulting in radiosensitivity (Fig. 7D). Collectively, our preclinical data show that activation of CLIP3 by glimepiride is a mechanism that counteracts radioresistance and improves survival of the GBM-bearing mice by targeting GSCs.

## Discussion

The standard of care for patients diagnosed with GBM has long entailed tumor resection, followed by radiotherapy and concomitant TMZ [24]. Despite this aggressive treatment regimen, long-term survival remains poor due to the persistence of radioresistant GSCs [43, 44]. Other approaches in development include immunotherapies, which have been gamechangers in heme malignancies but disappointed in solid tumors such as GBM [45], and oncolytic virus, which has shown promise when combined with immunotherapies [46]. However, directly targeting GSCs remains extremely challenging and there are still no approved drugs targeting GSCs [6].

Metabolic targeting of CSCs is being proposed as a new paradigm of cancer therapy [47]. To date, most clinical trials related to CSC metabolism have focused on targeting metabolic enzymes [17]. However, this approach is often toxic to normal cells, and accordingly, very few such drugs have been approved for clinical use [48]. In this study, we proposed to regulate glucose uptake by targeting GLUT3 trafficking in GBM cells. This strategy is in line with a recent study demonstrating that inhibiting Tubulin beta-4A chain (TUBB4) reduces levels of GLUT1, found to be overexpressed in astrocytoma, and inhibits self-renewal and tumor-initiating capacity in GSCs, although TUBB4 inhibition might well negatively impact on important cellular functions as well [49]. Interestingly, in contrast with GLUT1, GLUT3 expression is primarily elevated in GSCs and its expression highly correlates with poor survival in GBM [19], suggesting that targeting GLUT3 would more specifically disrupt GSCs. However, a recent study showed that a GLUT3 inhibitor induced cytotoxicity with the effective inhibition dose (50 μM) due to its general expression in neurons, indicating that directly inhibiting GLUT3 might induce serious side effects [50]. More broadly, glucose transporters are central to neuronal glucose uptake and brain metabolism, and targeted therapies will therefore need to take into account potential unwanted side effects [51]. In this context, the indirect mechanism we delineated here suggests that targeting CLIP3 to suppress GLUT3 cell surface trafficking would primarily effect CLIP3-expressing GBM cells such as GSCs.

GLUTs are continuously internalized by endocytosis and recycled to the cell membrane, so both transcriptional levels and recycling kinetics regulate the rate of glucose uptake [21]. To date, GLUT trafficking research has been heavily focused on GLUT4, which is mainly stimulated by insulin-derived signals up to 10 times that of baseline levels [20]. However, GLUT3 recycling and its cell surface level are known to be regulated by Rab11a, a member of the Rab family, mostly involved in vesicle trafficking including endosome recycling for several GLUTs [22]. According to our data, CLIP3 reduced GLUT3 membrane trafficking by disrupting the Rab11a-positive endosome, which was consistent with previous studies that CLIP3 contains a CAP-Gly domain, which is involved in vesicle and organelle transportation along the cytoskeletal network [52]. We anticipate that further studies by verifying binding partners involved in CLIP3-mediated GLUT3 translocation will identify cytoskeletal molecules for targeting GSCs.

Glimepiride is an FDA-approved oral drug for the treatment of type 2 diabetes mellitus with an acceptable side effect profile and classified as a second-generation sulfonylurea [41]. Sulfonylureas are organic compounds which close the K_ATP_ channels and open voltage-gated Ca^2+^ channels to increase the calcium influx, not only in pancreatic beta cells but also cells in other tissues, including the heart and brain [53]. Our in vitro data demonstrate that glimepiride significantly attenuates IR-induced gene expression changes, whereas inhibition of the K_ATP_ channel or glibenclamide did not. These different effects are likely explained by the different binding affinity of the two sulfonylureas. Because glimepiride has a 2.5 to 3-fold lower affinity to the K_ATP_ channel than glibenclamide, glimepiride might employ another molecular mechanism to regulate GSCs [54]. Although it is known that sulfonylureas do not effectively penetrate the blood-brain barrier (BBB), recent studies showed that glimepiride is able to cross the BBB and affect the brain under diabetic or stroke conditions where the BBB integrity is compromised [55]. Because BBB disruption is one of the common characteristics of GBM, these findings lend support to glimepiride having potential as a drug candidate for GBM and could even be orally administered as in diabetes treatment [56]. Indeed, our in vivo data indicate that glimepiride was delivered to the brain and acted as a radiosensitizer in GBM.

Our preclinical data demonstrate that glimepiride improves survival of GBM-bearing mice as effectively as TMZ when combined with IR even using the MGMT negative cell line, U87MG, with an efficacy more significant than predicted by our in vitro data. A recent clinical study showed that diabetic patients with GBM had poorer overall survival due to hyperglycemia [57]. Because glimepiride can induce insulin secretion to reduce blood glucose levels as well as CLIP3 activation, a decrease in blood glucose levels upon treatment of glimepiride might explain the significant in vivo efficacy in GBM mouse models. In addition, TMZ is highly toxic, whereas glimepiride side-effects are more manageable, and in combination with IR could therefore potentially achieve similar therapeutic efficacy with less burden to patients [58]. Further clinical studies are needed in order to optimize the dosage, the duration of the drug in human applications and the potential for using CLIP3 as a GBM biomarker.

## Conclusions

In this study, we identified the Spy1-CLIP3 axis as a critical regulator of GSC maintenance. We found that Spy1 was increased after IR and enhanced stemness by activating nuclear CDK2 in GBM cells. Mechanistically, CDK2 prevents NRF1 from binding to the promoter region of CLIP3, keeping CLIP3 transcription low after IR. Downregulation of CLIP3 in turn induces GLUT3 trafficking to cellular membranes and increases glycolytic activity, especially in GSCs. Importantly, our data demonstrate that the CLIP3 activator glimepiride targets GSC metabolism. Overall, this study suggests that radioresistant GBM cells that survive after radiotherapy exhibit increased stemness and glycolytic activity mediated by the Spy1-CLIP3 axis, and that glimepiride by activating CLIP3 can achieve high-efficiency radiosensitization with low toxicity (Fig. 7E). Clinical trials with glimepiride for GBM patients might improve survival, especially for patients who have suffered from recurrence after radiotherapy.

## Supplementary Information

**Additional file 1: Video S1**. GLUT3 trafficking in U87MG.

**Additional file 2: Video S2**. GLUT3 trafficking in response to IR in U87MG.

**Additional file 3: Video S3**. GLUT3 trafficking upon CLIP3 knockdown in U87MG.

**Additional file 4: Video S4**. GLUT3 trafficking in T98G.

**Additional file 5: Video S5**. GLUT3 trafficking in response to IR in T98G.

**Additional file 6: Video S6**. GLUT3 trafficking upon CLIP3 knockdown in T98G.

**Additional file 7: Table S1**. Overview of patient-derived primary glioblastoma cell lines.

**Additional file 8: Table S2**. Primers for determining levels of gene expression. **Table S3**. Sequences of the wild-type and mutant CLIP3 promoter linked to luciferase gene.

**Additional file 9: Figure S1**. Spy1 and CLIP3 expression in gliomas correlates with poor survival. **Figure S2**. GSCs are enriched by IR-induced CLIP3 downregulation in GBM cells. **Figure S3**. Spy1-CLIP3 axis contributes to metabolic shift toward glycolysis in patient-derived GSCs. **Figure S4**. Spy1-CLIP3 Axis Contributes to Metabolic Shift toward Glycolysis in GBM Cells. **Figure S5**. Glimepiride Disrupts GSC Maintenance by CLIP3 Activation. **Figure S6**. Glimepiride inhibits glucose uptake and lactate production. **Figure S7.** Glimepiride hardly changes the body weight of GBM-bearing mice.

## Data Availability

The accession number for the cDNA microarray analysis data reported in this paper is GEO: GSE117126.

## Abbreviations

BBB: Blood-brain barrier
CDKs: Cyclin-dependent kinases
CLIP3: CAP-Gly domain containing linker protein 3
CMap: The Connectivity Map
CSCs: Cancer stem cells
ECAR: Extracellular acidification rate
GBM: Glioblastoma
GLUT1: Glucose transporter 1
GLUT3: Glucose transporter 3
GLUT4: Glucose transporter 4
GSCs: Glioblastoma stem cells
IR: Ionizing radiation
K_ATP_: ATP-sensitive K^+^
MGMT: O_6_-Methylguanine-DNA Methyltransferase
NES: NESTIN
NRF1: Nuclear respiratory factor 1
OCR: Oxygen consumption rate
Rab11a: Ras-related protein Rab-11A
Spy1: Speedy/RINGO cell cycle regulator family member A
TBC1D4: TBC1 domain family member 4
TMZ: Temozolomide
TUBB4: Tubulin beta-4A chain

## References

[1] Weller M, Wick W, Aldape K, Brada M, Berger M, Pfister SM, Nishikawa R, Rosenthal M, Wen PY, Stupp R, Reifenberger G (2015). Glioma. Nat Rev Dis Primers.

[2] Alexander BM, Cloughesy TF (2017). Adult Glioblastoma. J Clin Oncol.

[3] Ostrom QT, Cioffi G, Gittleman H, Patil N, Waite K, Kruchko C, Barnholtz-Sloan JS (2019). CBTRUS statistical report: primary brain and other central nervous system tumors diagnosed in the United States in 2012-2016. Neuro-Oncology.

[4] Verhaak RG, Hoadley KA, Purdom E, Wang V, Qi Y, Wilkerson MD, Miller CR, Ding L, Golub T, Mesirov JP, Alexe G, Lawrence M, O'Kelly M, Tamayo P, Weir BA, Gabriel S, Winckler W, Gupta S, Jakkula L, Feiler HS, Hodgson JG, James CD, Sarkaria JN, Brennan C, Kahn A, Spellman PT, Wilson RK, Speed TP, Gray JW, Meyerson M, Getz G, Perou CM, Hayes DN, Cancer Genome Atlas Research Network (2010). Integrated genomic analysis identifies clinically relevant subtypes of glioblastoma characterized by abnormalities in PDGFRA, IDH1, EGFR, and NF1. Cancer Cell.

[5] Lathia JD, Mack SC, Mulkearns-Hubert EE, Valentim CL, Rich JN (2015). Cancer stem cells in glioblastoma. Genes Dev.

[6] Gimple RC, Bhargava S, Dixit D, Rich JN (2019). Glioblastoma stem cells: lessons from the tumor hierarchy in a lethal cancer. Genes Dev.

[7] Bao S, Wu Q, McLendon RE, Hao Y, Shi Q, Hjelmeland AB (2006). Glioma stem cells promote radioresistance by preferential activation of the DNA damage response. Nature.

[8] Beier D, Schulz JB, Beier CP (2011). Chemoresistance of glioblastoma cancer stem cells--much more complex than expected. Mol Cancer.

[9] Saygin C, Matei D, Majeti R, Reizes O, Lathia JD (2019). Targeting Cancer Stemness in the clinic: from hype to Hope. Cell Stem Cell.

[10] Lubanska D, Market-Velker BA, de Carvalho AC, Mikkelsen T, Fidalgo da Silva E, Porter LA (2014). The cyclin-like protein Spy1 regulates growth and division characteristics of the CD133+ population in human glioma. Cancer Cell.

[11] Ding Z, Liu Y, Yao L, Wang D, Zhang J, Cui G, Yang X, Huang X, Liu F, Shen A (2015). Spy1 induces de-ubiquitinating of RIP1 arrest and confers glioblastoma's resistance to tumor necrosis factor (TNF-α)-induced apoptosis through suppressing the association of CLIPR-59 and CYLD. Cell Cycle.

[12] Du K, Yingmin S (2015). ClipR-59 plays a critical role in the regulation of body glucose homeostasis. Adipocyte.

[13] Ren W, Cheema S, Du K (2012). The association of ClipR-59 protein with AS160 modulates AS160 protein phosphorylation and adipocyte Glut4 protein membrane translocation. J Biol Chem.

[14] Yadav UP, Singh T, Kumar P, Sharma P, Kaur H, Sharma S, Singh S, Kumar S, Mehta K (2020). Metabolic adaptations in Cancer stem cells. Front Oncol.

[15] Vander Heiden MG, Cantley LC, Thompson CB (2009). Understanding the Warburg effect: the metabolic requirements of cell proliferation. Science.

[16] Mergenthaler P, Lindauer U, Dienel GA, Meisel A (2013). Sugar for the brain: the role of glucose in physiological and pathological brain function. Trends Neurosci.

[17] Luengo A, Gui DY, Vander Heiden MG (2017). Targeting metabolism for Cancer therapy. Cell Chem Biol.

[18] Son B, Lee S, Kim H, Kang H, Jeon J, Jo S, Seong KM, Lee SJ, Youn HS, Youn BH (2020). Decreased FBP1 expression rewires metabolic processes affecting aggressiveness of glioblastoma. Oncogene.

[19] Flavahan WA, Wu Q, Hitomi M, Rahim N, Kim Y, Sloan AE, Weil RJ, Nakano I, Sarkaria JN, Stringer BW, Day BW, Li M, Lathia JD, Rich JN, Hjelmeland AB (2013). Brain tumor initiating cells adapt to restricted nutrition through preferential glucose uptake. Nat Neurosci.

[20] Leto D, Saltiel AR (2012). Regulation of glucose transport by insulin: traffic control of GLUT4. Nat Rev Mol Cell Biol.

[21] Shinde SR, Maddika S (2017). PTEN regulates glucose transporter recycling by impairing SNX27 Retromer assembly. Cell Rep.

[22] McClory H, Williams D, Sapp E, Gatune LW, Wang P, DiFiglia M, Li X (2014). Glucose transporter 3 is a rab11-dependent trafficking cargo and its transport to the cell surface is reduced in neurons of CAG140 Huntington's disease mice. Acta Neuropathol Commun.

[23] Roy S, Leidal AM, Ye J, Ronen SM, Debnath J (2017). Autophagy-Dependent Shuttling of TBC1D5 Controls Plasma Membrane Translocation of GLUT1 and Glucose Uptake. Mol Cell.

[24] Stupp R, Mason WP, van den Bent MJ, Weller M, Fisher B, Taphoorn MJ (2005). Radiotherapy plus concomitant and adjuvant temozolomide for glioblastoma. N Engl J Med.

[25] Sitbon Sitruk L, Sanson M, Prades M, Lefebvre G, Schubert B (2010). Poirot C: [unknown gonadotoxicity chemotherapy and preservation of fertility: example of Temozolomide]. Gynecol Obstet Fertil.

[26] Kourelis TV, Buckner JC, Gangat N, Patnaik MM (2015). Temozolomide induced bone marrow suppression--a single institution outcome analysis and review of the literature. Am J Hematol.

[27] Lee SY (2016). Temozolomide resistance in glioblastoma multiforme. Genes Dis.

[28] Chua J, Nafziger E, Leung D (2019). Evidence-Based Practice: Temozolomide Beyond Glioblastoma. Curr Oncol Rep.

[29] Touat M, Idbaih A, Sanson M, Ligon KL (2017). Glioblastoma targeted therapy: updated approaches from recent biological insights. Ann Oncol.

[30] Xue H, Li J, Xie H, Wang Y (2018). Review of drug repositioning approaches and resources. Int J Biol Sci.

[31] Park G, Son B, Kang J, Lee S, Jeon J, Kim JH, Yi GR, Youn HS, Moon C, Nam SY, Youn BH (2019). LDR-induced miR-30a and miR-30b target the PAI-1 pathway to control adverse effects of NSCLC radiotherapy. Mol Ther.

[32] Kim W, Youn H, Lee S, Kim E, Kim D, Sub Lee J, Lee JM, Youn BH (2018). RNF138-mediated ubiquitination of rpS3 is required for resistance of glioblastoma cells to radiation-induced apoptosis. Exp Mol Med.

[33] Yang HJ, Youn H, Seong KM, Jin YW, Kim J, Youn B (2013). Phosphorylation of ribosomal protein S3 and antiapoptotic TRAF2 protein mediates radioresistance in non-small cell lung cancer cells. J Biol Chem.

[34] Hu Y, Smyth GK (2009). ELDA: extreme limiting dilution analysis for comparing depleted and enriched populations in stem cell and other assays. J Immunol Methods.

[35] Kim W, Youn H, Kang C, Youn B (2015). Inflammation-induced radioresistance is mediated by ROS-dependent inactivation of protein phosphatase 1 in non-small cell lung cancer cells. Apoptosis.

[36] Son B, Kwon T, Lee S, Han I, Kim W, Youn H, Youn BH (2017). CYP2E1 regulates the development of radiation-induced pulmonary fibrosis via ER stress- and ROS-dependent mechanisms. Am J Physiol Lung Cell Mol Physiol.

[37] Bowman RL, Wang Q, Carro A, Verhaak RG, Squatrito M (2017). GlioVis data portal for visualization and analysis of brain tumor expression datasets. Neuro-Oncology.

[38] Palmer N, Talib SZA, Ratnacaram CK, Low D, Bisteau X, Lee JHS, Pfeiffenberger E, Wollmann H, Tan JHL, Wee S, Sobota R, Gunaratne J, Messerschmidt DM, Guccione E, Kaldis P (2019). CDK2 regulates the NRF1/Ehmt1 axis during meiotic prophase I. J Cell Biol.

[39] Wang J, Yang T, Xu G, Liu H, Ren C, Xie W, Wang M (2016). Cyclin-dependent kinase 2 promotes tumor proliferation and induces radio resistance in glioblastoma. Transl Oncol.

[40] Subramanian A, Narayan R, Corsello SM, Peck DD, Natoli TE, Lu X (2017). A Next Generation Connectivity Map: L1000 Platform and the First 1,000,000 Profiles. Cell.

[41] Basit A, Riaz M, Fawwad A (2012). Glimepiride: evidence-based facts, trends, and observations (GIFTS). [corrected]. Vasc Health Risk Manag.

[42] Song DK, Ashcroft FM (2001). Glimepiride block of cloned beta-cell, cardiac and smooth muscle K (ATP) channels. Br J Pharmacol.

[43] Carruthers RD, Ahmed SU, Ramachandran S, Strathdee K, Kurian KM, Hedley A, Gomez-Roman N, Kalna G, Neilson M, Gilmour L, Stevenson KH, Hammond EM, Chalmers AJ (2018). Replication stress drives constitutive activation of the DNA damage response and Radioresistance in glioblastoma stem-like cells. Cancer Res.

[44] Shi Y, Guryanova OA, Zhou W, Liu C, Huang Z, Fang X, et al. Ibrutinib inactivates BMX-STAT3 in glioma stem cells to impair malignant growth and radioresistance. Sci Transl Med. 2018;10(443):eaah6816. 10.1126/scitranslmed.aah6816. 10.1126/scitranslmed.aah6816 PMC6431250 29848664

[45] Pearson JRD, Cuzzubbo S, McArthur S, Durrant LG, Adhikaree J, Tinsley CJ, Pockley AG, McArdle SEB (2020). Immune escape in glioblastoma Multiforme and the adaptation of immunotherapies for treatment. Front Immunol.

[46] Zhang Q, Liu F (2020). Advances and potential pitfalls of oncolytic viruses expressing immunomodulatory transgene therapy for malignant gliomas. Cell Death Dis.

[47] Chae YC, Kim JH (2018). Cancer stem cell metabolism: target for cancer therapy. BMB Rep.

[48] Zhai L, Bell A, Ladomersky E, Lauing KL, Bollu L, Sosman JA, Zhang B, Wu JD, Miller SD, Meeks JJ, Lukas RV, Wyatt E, Doglio L, Schiltz GE, McCusker RH, Wainwright DA (2020). Immunosuppressive IDO in Cancer: mechanisms of action, animal models, and targeting strategies. Front Immunol.

[49] Guda MR, Labak CM, Omar SI, Asuthkar S, Airala S, Tuszynski J (2019). GLUT1 and TUBB4 in Glioblastoma Could be Efficacious Targets. Cancers (Basel)..

[50] Libby CJ, Zhang S, Benavides GA, Scott SE, Li Y, Redmann M, Tran AN, Otamias A, Darley-Usmar V, Napierala M, Zhang J, Augelli-Szafran CE, Zhang W, Hjelmeland AB (2018). Identification of compounds that decrease glioblastoma growth and glucose uptake in vitro. ACS Chem Biol.

[51] Ancey PB, Contat C, Meylan E (2018). Glucose transporters in cancer - from tumor cells to the tumor microenvironment. FEBS J.

[52] Li S, Finley J, Liu ZJ, Qiu SH, Chen H, Luan CH, Carson M, Tsao J, Johnson D, Lin G, Zhao J, Thomas W, Nagy LA, Sha B, DeLucas LJ, Wang BC, Luo M (2002). Crystal structure of the cytoskeleton-associated protein glycine-rich (CAP-Gly) domain. J Biol Chem.

[53] Kalra S, Aamir AH, Raza A, Das AK, Azad Khan AK, Shrestha D (2015). Place of sulfonylureas in the management of type 2 diabetes mellitus in South Asia: a consensus statement. Indian J Endocrinol Metab.

[54] Briscoe VJ, Griffith ML, Davis SN (2010). The role of glimepiride in the treatment of type 2 diabetes mellitus. Expert Opin Drug Metab Toxicol.

[55] Szeto V, Chen NH, Sun HS, Feng ZP (2018). The role of K (ATP) channels in cerebral ischemic stroke and diabetes. Acta Pharmacol Sin.

[56] Arvanitis CD, Ferraro GB, Jain RK (2020). The blood-brain barrier and blood-tumour barrier in brain tumours and metastases. Nat Rev Cancer.

[57] Barami K, Lyon L, Conell C (2017). Type 2 diabetes mellitus and glioblastoma Multiforme-assessing risk and survival: results of a large retrospective study and systematic review of the literature. World Neurosurg.

[58] Amate JM, Lopez-Cuadrado T, Almendro N, Bouza C, Saz-Parkinson Z, Rivas-Ruiz R, Gonzalez-Canudas J (2015). Effectiveness and safety of glimepiride and iDPP4, associated with metformin in second line pharmacotherapy of type 2 diabetes mellitus: systematic review and meta-analysis. Int J Clin Pract.

