# Supplementary material for: Downregulated CLIP3 induces radioresistance by enhancing stemness and glycolytic flux in glioblastoma
Source: J Exp Clin Cancer Res. 2021 Sep 6;40:282. doi: 10.1186/s13046-021-02077-4 (PMC8420000; doi:10.1186/s13046-021-02077-4)
Supplement: Supplementary file 8 — Additional file 8: Table S2. Primers for determining levels of gene expression. Table S3. Sequences of the wild-type and mutant CLIP3 promoter linked to luciferase gene. [file 13046_2021_2077_MOESM8_ESM.docx]

**Additional file 8. Table S2-S3**

**Table S2.** Primers for determining levels of gene expression

| Gene name | Forward primer | Reverse primer |
| --- | --- | --- |
| *Spy1*  *CLIP3*  *NRF1*  *NANOG*  *OCT4*  *GLUT1*  *GLUT3* | 5′-TTG TGA GGA GGT TAT GGC CAT T-3′  5′-CTC CAC GTG CAG TGA CTT CAA-3′  5′- CCA CAG GCA GAT GAA TGT CTT G -3′  5′- GAA ATA CCT CAG CCT CCA GC -3′  5′- TCT CCC ATG CAT TCA AAC TGA G -3′  5′-ATC GTG GCC ATC TTT GGC TTT GTG-3′  5′-AGC TCT CTG GGA TCA ATG CTG TGT-3′ | 5′-GCA GCT GAA CTT CAT CTC TGT TGT AG-3′  5′-AGG CTG GAA GCA GCG ATG T-3′  5′- TCC TGG GAA GGA GAG GAG ATG -3′  5′- GCG TCA CAC CAT TGC TAT TC -3′  5′- CCT TTG TGT TCC CAA TTC CTT C -3′  5′-CTG GAA GCA CAT GCC CAC AAT GAA-3′  5′-ATG GTG GCA TAG ATG GGC TCT TGA-3′ |

**Table S3.** Sequences of the wild-type and mutant CLIP3 promoter linked to luciferase gene

| CLIP3 promoter | NRF1 consensus sequence [distance from transcription start site] |
| --- | --- |
| Wild-type  Mutant | [-24] GCGCATGCGCA [-14]  [-24] GCAAATTTGCA [-14] |
