# Supplementary material for: Downregulated CLIP3 induces radioresistance by enhancing stemness and glycolytic flux in glioblastoma
Source: J Exp Clin Cancer Res. 2021 Sep 6;40:282. doi: 10.1186/s13046-021-02077-4 (PMC8420000; doi:10.1186/s13046-021-02077-4)
Supplement: Supplementary file 9 — Additional file 9: Figure S1. Spy1 and CLIP3 expression in gliomas correlates with poor survival. Figure S2. GSCs are enriched by IR-induced CLIP3 downregulation in GBM cells. Figure S3. Spy1-CLIP3 axis contributes to metabolic shift toward glycolysis in patient-derived GSCs. Figure S4. Spy1-CLIP3 Axis Contributes to Metabolic Shift toward Glycolysis in GBM Cells. Figure S5. Glimepiride Disrupts GSC Maintenance by CLIP3 Activation. Figure S6. Glimepiride inhibits glucose uptake and lactate production. Figure S7. Glimepiride hardly changes the body weight of GBM-bearing mice. [file 13046_2021_2077_MOESM9_ESM.docx]

**Additional file 9. Figure S1-S7**

**
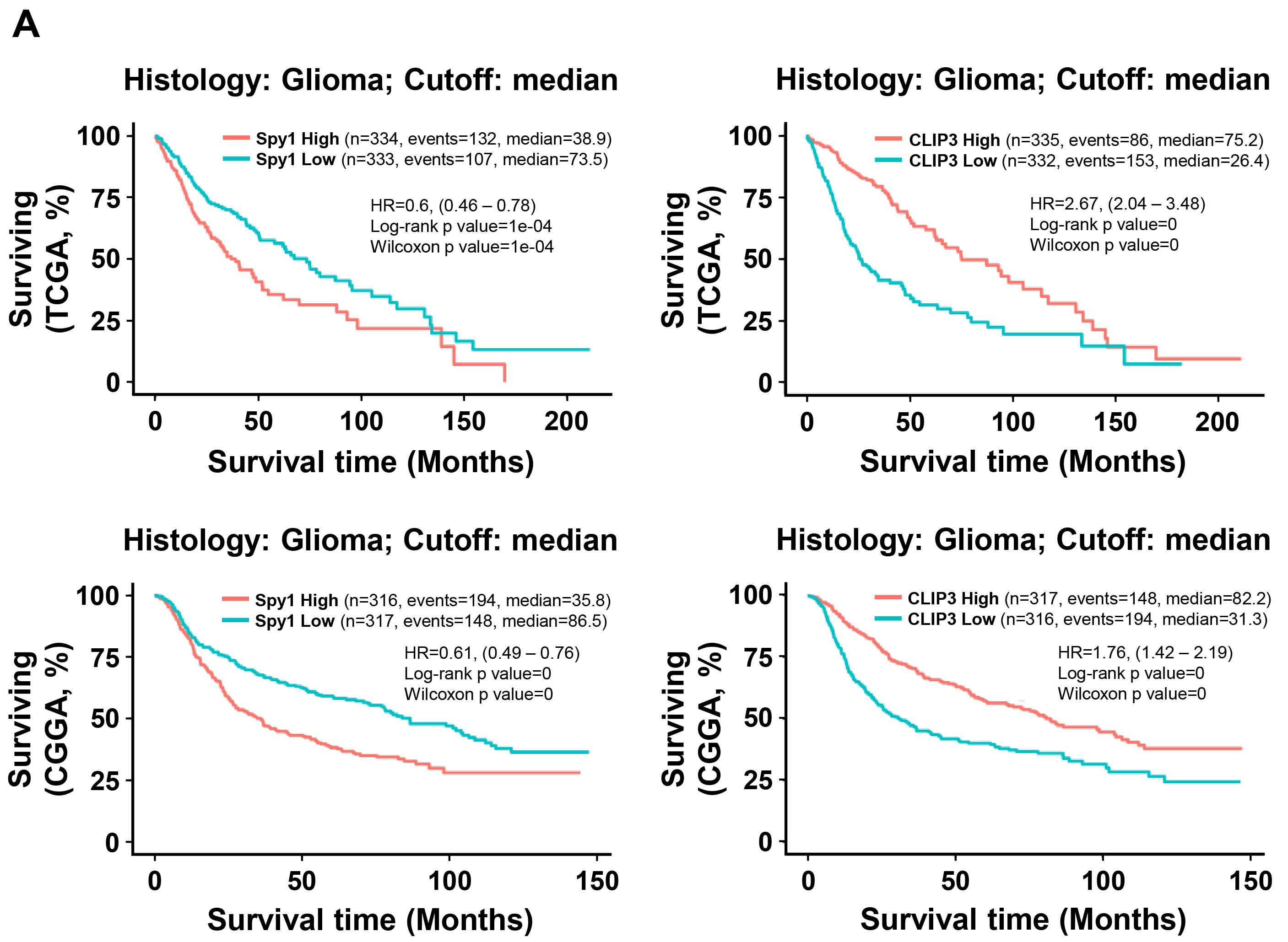
**

**Figure S1. Spy1 and CLIP3 expression in gliomas correlates with poor survival**

(A) Survival rate calculated by the Kaplan-Meier survival curve in glioma patients separated according to median expression level of Spy1 and CLIP3.

**
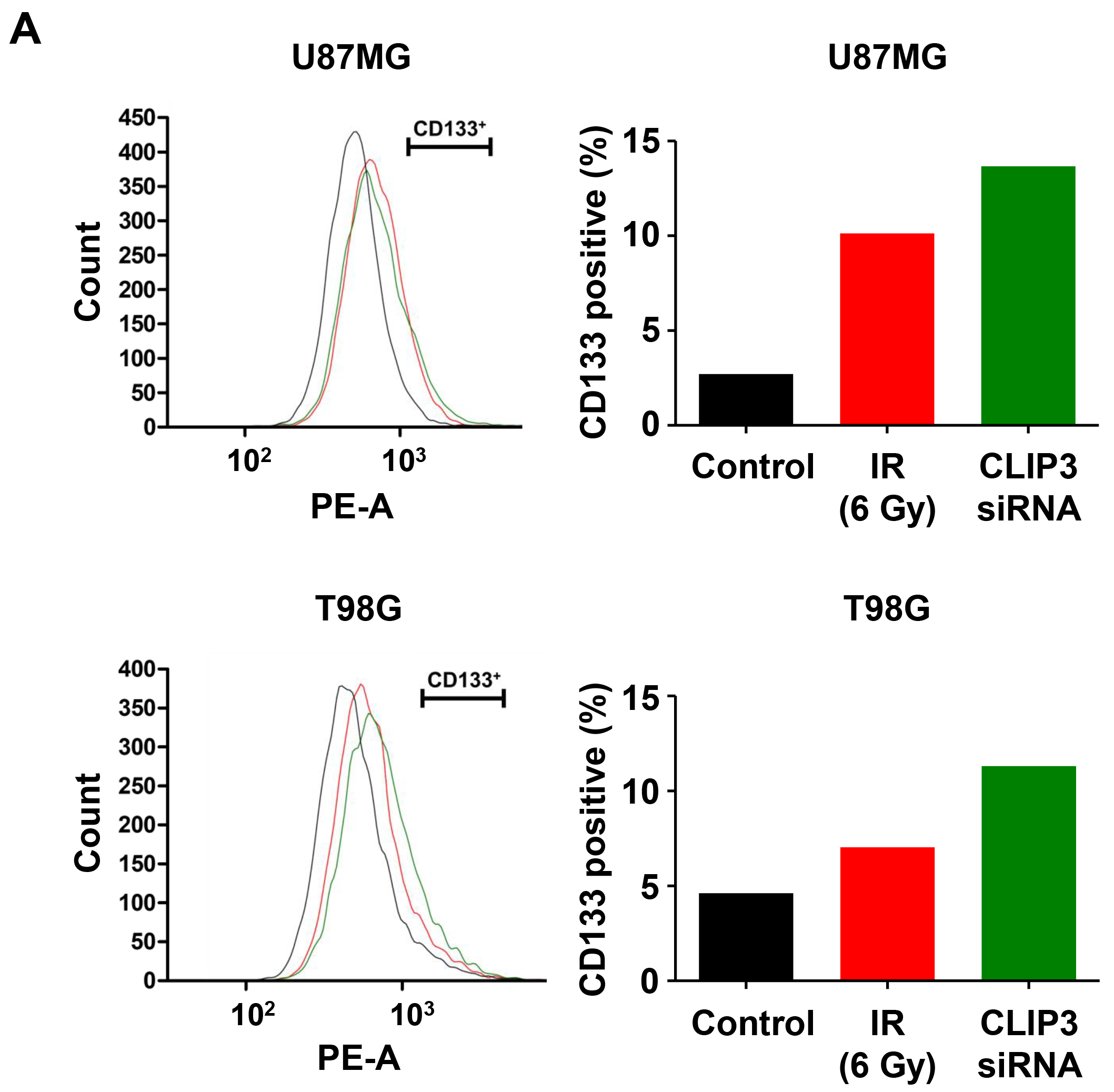
**

**Figure S2. GSCs are enriched by IR-induced CLIP3 downregulation in GBM cells**

(A) Percentages of CD133^+^ cells were measured by flow cytometry after IR (6 Gy) or CLIP3 siRNA treatment in U87MG and T98G cells.

**
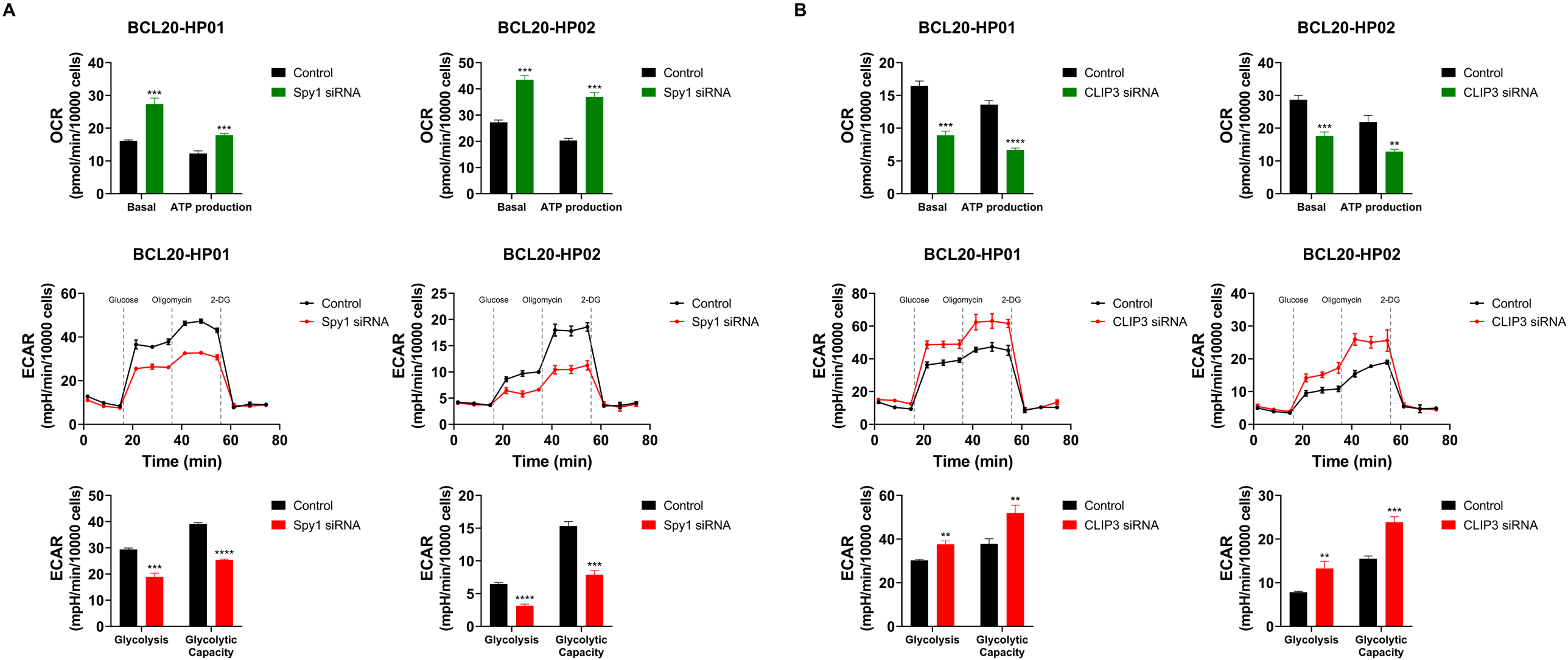
**

**Figure S3. Spy1-CLIP3 axis contributes to metabolic shift toward glycolysis in patient-derived GSCs**

(A, B) OCR and ECAR were measured by Seahorse analyzer in BCL20-HP01 and BCL20-HP02 cells. Basal OCR indicates the basal level of oxygen consumption and ATP production indicates the decrease in oxygen consumption rate upon injection of oligomycin, which represents the portion of basal respiration that was being used to drive ATP production. Glycolysis indicates the generation of lactate upon glucose addition and glycolytic capacity indicates the maximum capacity of lactate generation upon inhibition of oxidative phosphorylation. OCR and ECAR upon transfection of Spy1 siRNA (A) or CLIP3 siRNA (B). **p < 0.01, ***p < 0.001, ****p < 0.0001 with unpaired t-test.


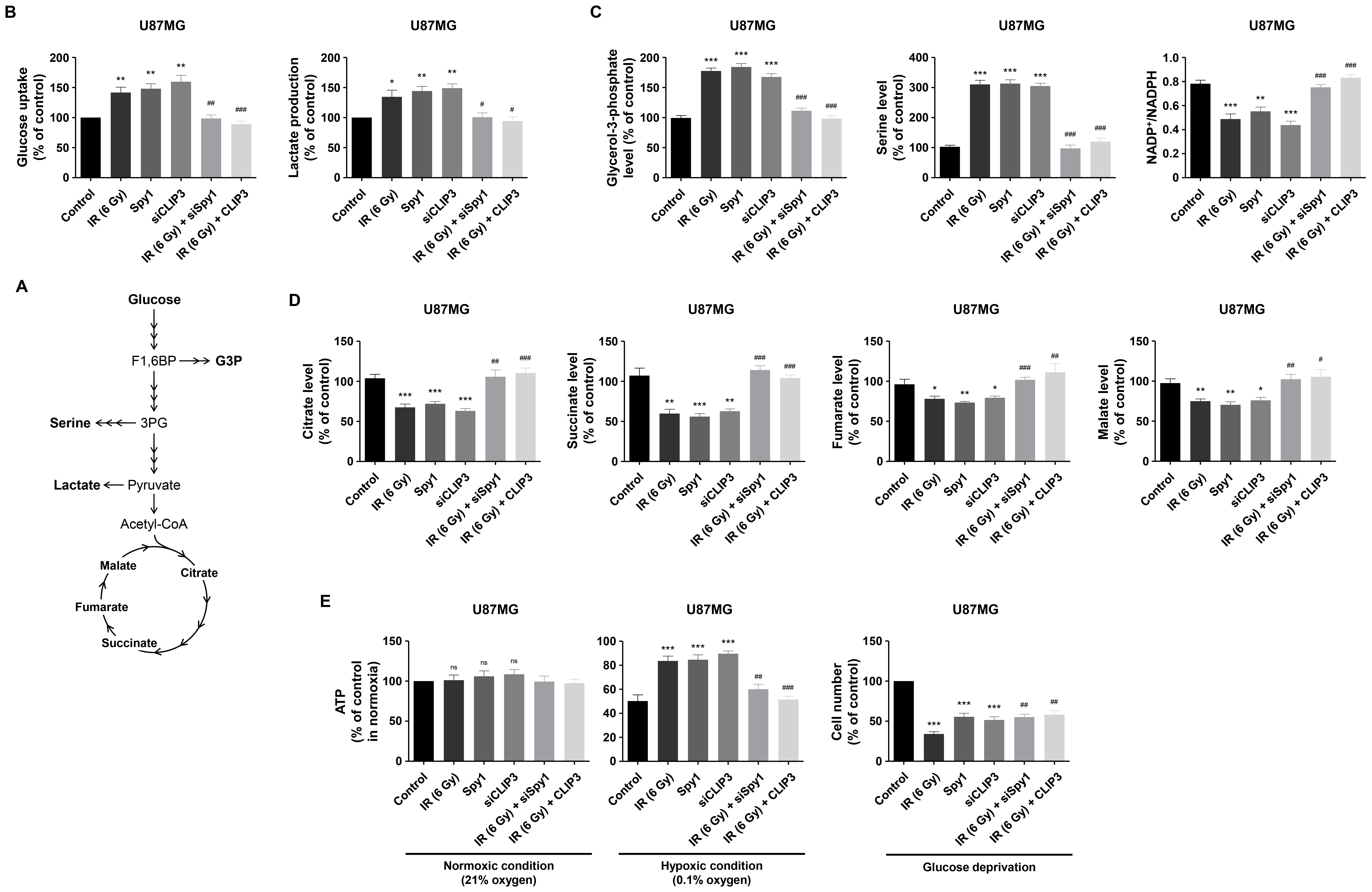


**Figure S4. Spy1-CLIP3 Axis Contributes to Metabolic Shift toward Glycolysis in GBM Cells**

(A) Brief schematic diagram illustrating glycolysis and citric acid cycle. F1,6BP, fructose 1,6-bisphosphate; G3P, glycerol-3-phosphate; 3PG, 3-phosphoglycerate. (B) Glucose uptake and lactate production were measured upon treatment of IR (6 Gy), Spy1 gene, CLIP3 siRNA, IR with Spy1 siRNA, or IR with CLIP3 gene. (C, D) Metabolic intermediates were measured upon treatment of IR (6 Gy), Spy1 gene, CLIP3 siRNA, IR with Spy1 siRNA, or IR with CLIP3 gene. (E) ATP production in normoxic condition (21% oxygen) and hypoxic condition (0.1% oxygen) was measured upon treatment of IR (6 Gy), Spy1 gene, CLIP3 siRNA, IR with Spy1 siRNA, or IR with CLIP3 gene (left panel). Cell number under glucose deprivation was counted upon treatment of IR (6 Gy), Spy1 gene, CLIP3 siRNA, IR with Spy1 siRNA, or IR with CLIP3 gene (right panel). Statistical analysis was performed with one-way ANOVA plus a Tukey’s multiple comparisons test. **p* < 0.05, ***p* < 0.01, ****p* < 0.001 (compared to control). ^#^*p* < 0.05, ^##^*p* < 0.01, ^###^*p* < 0.001 (compared to IR).


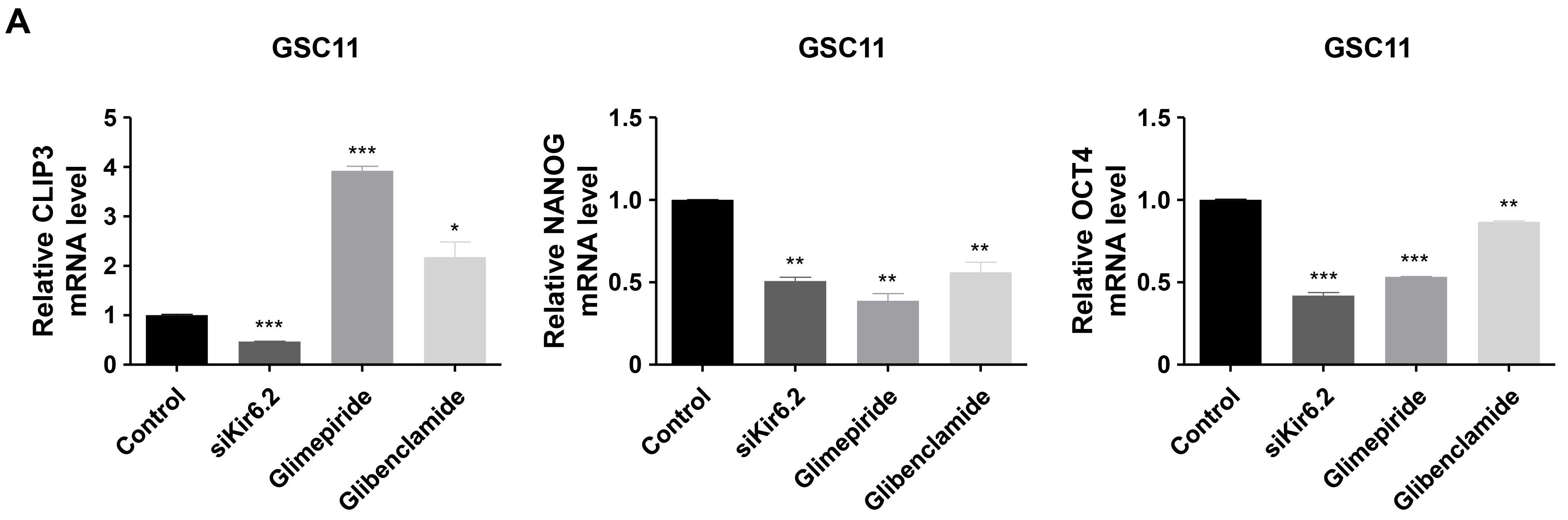


**Figure S5. Glimepiride Disrupts GSC Maintenance by CLIP3 Activation**

(A) mRNA levels of CLIP3, NANOG, and OCT4 were analyzed by real-time qRT-PCR upon treatment of Kir6.2 siRNA, glimepiride (1 μM), or glibenclamide (1 μM) in GSC11 cells. Statistical analysis was performed with one-way ANOVA plus a Tukey’s multiple comparisons test. **p* < 0.05, ***p* < 0.01, ****p* < 0.001.


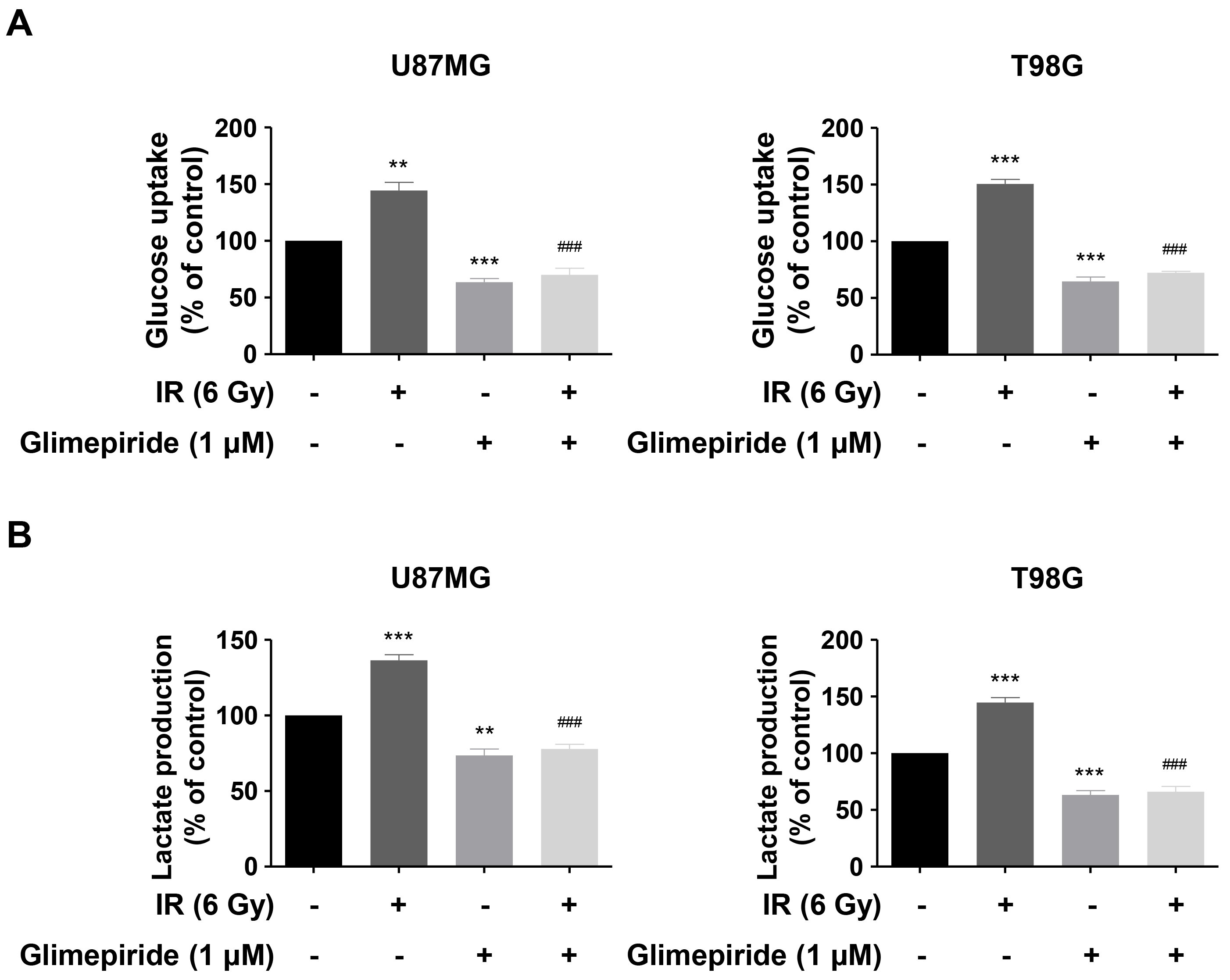


**Figure S6. Glimepiride inhibits glucose uptake and lactate production**

(A) Glucose uptake was measured upon treatment of IR (6 Gy), glimepiride (1 μM), or IR with glimepiride. (B) Lactate production was measured upon treatment of IR (6 Gy), glimepiride (1 μM), or IR with glimepiride. Statistical analysis was performed with one-way ANOVA plus a Tukey’s multiple comparisons test. **p* < 0.05, ***p* < 0.01, ****p* < 0.001 (compared to control). ^#^*p* < 0.05, ^##^*p* < 0.01, ^###^*p* < 0.001 (compared to IR).


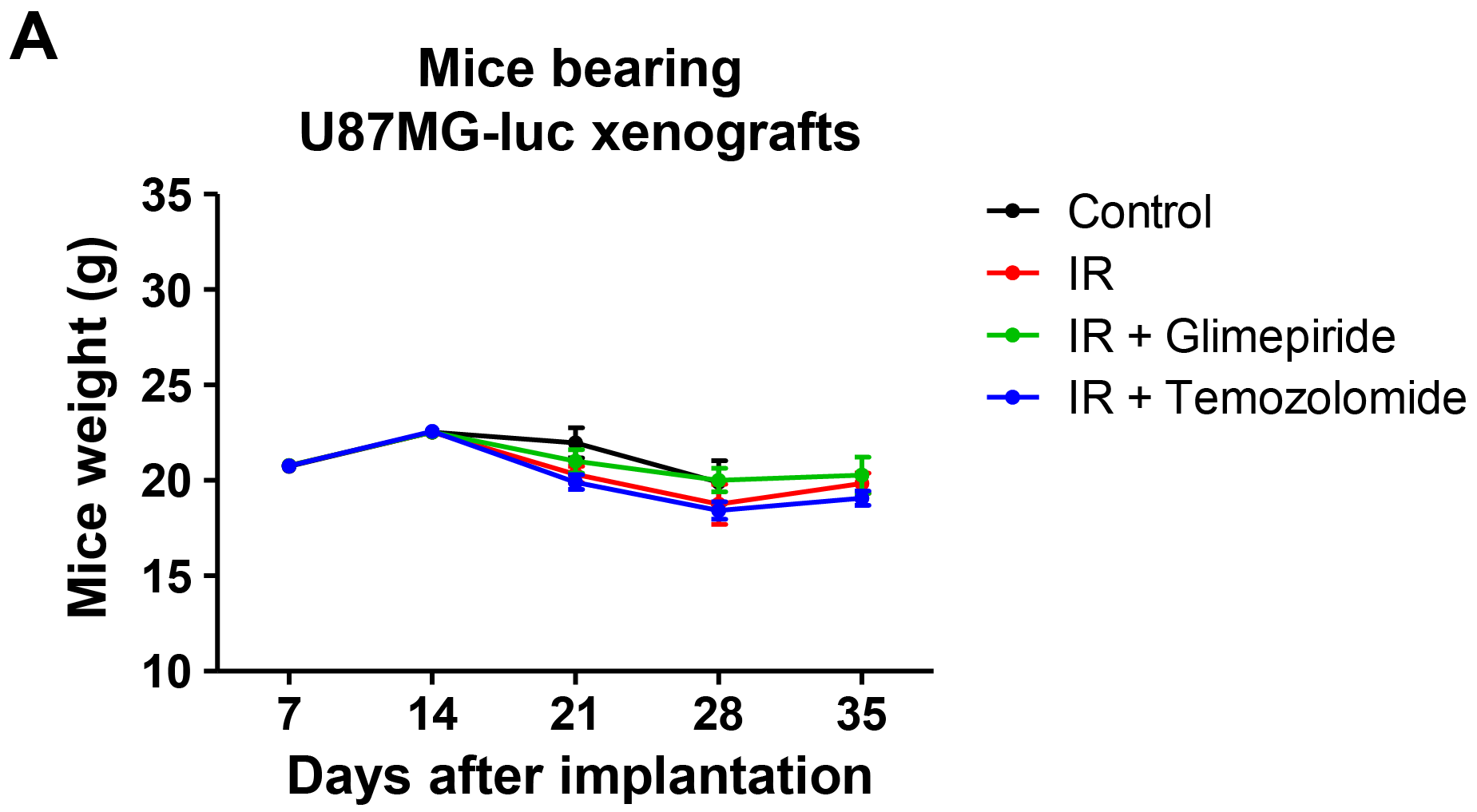


**Figure S7.** **Glimepiride hardly changes the body weight of GBM-bearing mice**

(A) Body weight of mice bearing U87MG-luciferase xenografts control, treated with IR, IR with glimepiride, or IR with temozolomide.
